# Proximal methylation features associated with nonrandom changes in gene body methylation

**DOI:** 10.1186/s13059-017-1206-2

**Published:** 2017-04-26

**Authors:** Colette L. Picard, Mary Gehring

**Affiliations:** 1 Whitehead Institute for Biomedical Research, Cambridge, MA 02142 USA; 2 Computational and Systems Biology Graduate Program, Massachusetts Institute of Technology, Cambridge, MA 02139 USA; 3 Department of Biology, Massachusetts Institute of Technology, Cambridge, MA 02139 USA

**Keywords:** DNA methylation, Epigenetic inheritance, Gene body methylation, *Arabidopsis*

## Abstract

**Background:**

Gene body methylation at CG dinucleotides is a widely conserved feature of methylated genomes but remains poorly understood. The *Arabidopsis thaliana* strain Cvi has depleted gene body methylation relative to the reference strain Col. Here, we leverage this natural epigenetic difference to investigate gene body methylation stability.

**Results:**

Recombinant inbred lines derived from Col and Cvi were used to examine the transmission of distinct gene body methylation states. The vast majority of genic CG methylation patterns are faithfully transmitted over nine generations according to parental genotype, with only 1–4% of CGs either losing or gaining methylation relative to the parent. Genic CGs that fail to maintain the parental methylation state are shared among independent lines, suggesting that these are not random occurrences. We use a logistic regression framework to identify features that best predict sites that fail to maintain parental methylation state. Intermediate levels of CG methylation around a dynamic CG site and high methylation variability across many *A. thaliana* strains at that site are the strongest predictors. These data suggest that the dynamic CGs we identify are not specific to the Col–Cvi recombinant inbred lines but have an epigenetic state that is inherently less stable within the *A. thaliana* species. Extending this, variably methylated genic CGs in maize and *Brachypodium distachyon* are also associated with intermediate local CG methylation.

**Conclusions:**

These results provide new insights into the features determining the inheritance of gene body methylation and demonstrate that two different methylation equilibria can be maintained within single individuals.

**Electronic supplementary material:**

The online version of this article (doi:10.1186/s13059-017-1206-2) contains supplementary material, which is available to authorized users.

## Background

Gene body methylation is a widely conserved feature of methylated eukaryotic genomes and has been described in plants [1–5], various insects [6], mammals, including humans [7, 8], and others [9, 10]. Body-methylated genes, which make up about 30% of genes in *A. thaliana* [2, 3], are moderately expressed [2, 3, 7, 10, 11], longer than unmethylated genes [3, 4], usually present in a single copy in the genome [12], and slowly evolving [4, 13]. Levels of gene body methylation are well conserved between orthologs in related species, such as *Brachypodium distachyon*, rice, and maize [14], and honeybee and the parasitoid wasp *Nasonia vitripennis* [13]. Together, these observations suggest that gene body methylation levels might have been evolutionarily selected for at some loci for an as-yet undetermined function. While some evidence suggests that gene body methylation can affect gene expression [15], regulate splicing [16], or prevent aberrant transcription initiation [17], most studies find little evidence of a causal relationship between gene body methylation and gene expression in plants [2, 18–20]. This has led to the suggestion that gene body methylation is merely a byproduct of other methylation pathways [20] or transcription [21].

The origin of gene body methylation remains unclear. Gene body methylation only occurs at cytosines in the CG context. In plants, this is in contrast to methylation elsewhere in the genome, which is found at cytosines in the CG, CHG, and CHH sequence contexts. Methylation co-occurring in all three contexts is often associated with repetitive sequences and transcriptional silencing and is established by the RNA-directed DNA methylation (RdDM) pathway. Non-CG methylation is maintained by RdDM, CMT3, and CMT2 (reviewed in [22]). The absence of non-CG methylation in gene bodies [1–3] suggests that these pathways do not presently target genes. CG methylation is maintained by the maintenance methyltransferase MET1, which methylates the new strand of replicated DNA based on the pattern of methylation on the old strand [22]. Loss of MET1 leads to almost complete loss of gene body methylation, which often does not return even many generations after functional MET1 is reintroduced [23–25].

The lack of genetic or molecular resources for targeted alteration of gene body methylation has made investigating this type of methylation in isolation difficult. Mutants that lack gene body methylation, such as *met1*, also lack CG and non-CG methylation throughout the genome and have pleiotropic phenotypes [26, 27]. However, *Arabidopsis thaliana* is distributed worldwide and exhibits considerable natural epigenetic variation [12, 19]. We previously showed that an *A. thaliana* strain from the Cape Verde Islands (Cvi) has approximately half as much genic CG methylation as the reference strains Col and L*er*, but similar levels of non-genic methylation [28], making it a potentially powerful tool for specifically studying gene body methylation. Here, we further characterize gene body methylation in Cvi and profile DNA methylation in ten Col–Cvi recombinant inbred lines (RILs) [29] to examine how different methylation states are transmitted to progeny. While most genes in the RILs had CG methylation similar to the parent line from which the gene was inherited, individual genic CGs gained or lost methylation relative to the parent line at a low rate (1–4%) in each RIL. We examined whether sequence composition, sequence motifs, methylation patterns, small RNAs, or various other features were associated with these dynamically methylated sites. Dynamic cytosines were associated with several distinct local methylation features. Using a regression approach, we found that intermediate local CG methylation and variable methylation across *A. thaliana* strains were the best predictors of dynamic CG sites in the RILs.

## Results

### Cvi genes lack methylation at a subset of CG sites

**Fig. 1:**
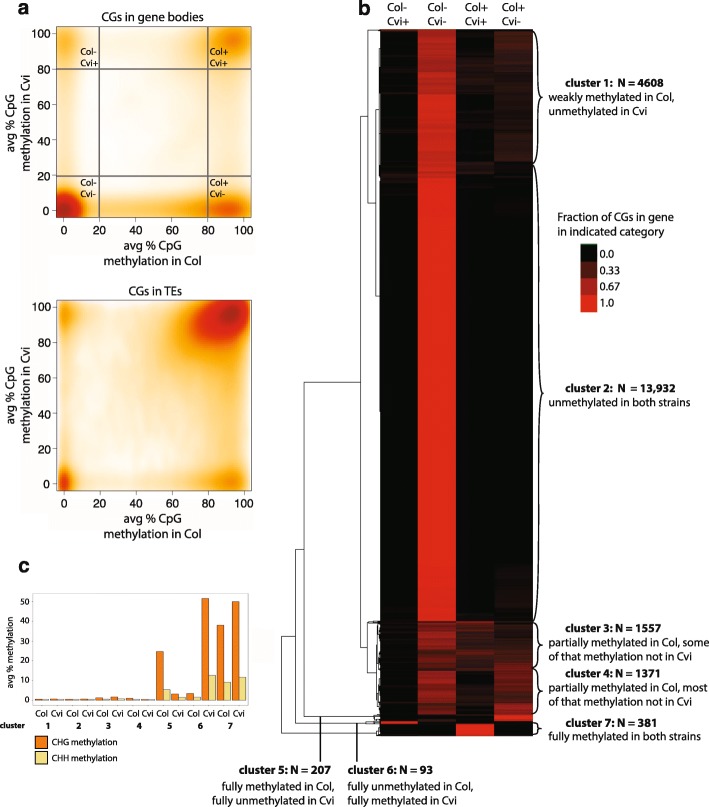
Gene body methylation at individual CGs in Col and Cvi. **a** Smoothed scatterplot of average CG methylation in Col versus Cvi for individual CGs within gene bodies (*top*) or transposable elements (TEs; *bottom*). CGs in the four corners of the *top plot* are used in **b**. Number of cytosines in each corner: *Col−, Cvi −* = 673,701; *Col−, Cvi +* = 10,500; *Col+, Cvi −* = 79,667; *Col+, Cvi +* = 51,575. Total CGs in genic plot = 867,234. **b** Hierarchical clustering of the 22,149 genes with at least ten CGs classified into any of the four categories in **a**. *Rows* represent genes, *columns* represent the four categories in **a**, and *color* represents fraction of CGs in each of the four categories for each gene. Genes were grouped into seven clusters. **c** Average non-CG methylation levels among genes in different clusters from **b**

To better characterize the differences in methylation between Col and Cvi, we performed whole-genome bisulfite sequencing of leaf DNA (Additional file 1: Table S1). Cvi lacked methylation at a subset of genic CG dinucleotides that were methylated in Col (Fig. 1a), whereas transposable element (TE) methylation and non-CG methylation were similar in both strains (Fig. 1a; Additional file 1: Figure S1). For the purposes of this study, genic CG dinucleotides are defined as all CGs between transcriptional start sites and transcriptional termination sites that do not overlap an annotated TE. The majority (77.7%) of genic CG sites lacked methylation in both strains (defined as ≤20% methylation), whereas 5.9% were highly methylated in both strains (defined as ≥80% methylation). By contrast, 9.2% were methylated in Col but not Cvi, and only 1.2% were methylated in Cvi but not Col (Fig. 1a). To compare methylation between Col and Cvi at the gene level, we calculated the fraction of CGs in each of these four categories for each gene and performed hierarchical clustering (Fig. 1b; Additional file 1: Figure S1). Most genes had little to no gene body methylation in either strain, consistent with previous reports [2, 3]. A set of 381 genes (cluster 7 in Fig. 1b) were highly CG methylated in both strains. These genes were also associated with significant non-CG methylation (Fig. 1c) and are likely RdDM targets. Two small groups of genes had high CG methylation specific to one strain (clusters 5 and 6), as well as non-CG methylation in the methylated strain (Fig. 1c). The presence of non-CG methylation suggests that these genes are strain-specific RdDM targets. These genes also had higher bisulfite sequencing read coverage in the strain with non-CG methylation (Additional file 1: Figure S1). Although preferential amplification of methylated DNA during bisulfite sequencing could explain some of these differences [30], these results could also indicate that there are strain-specific copy number increases at these loci, which would be consistent with their methylation profile since repetitive sequences are often RdDM targets. The 93 genes methylated specifically in Cvi (cluster 6) were strongly enriched for F-box genes (enrichment score 23.14, adj *p* value 9.2 × 10^–30^) [31], one of the largest and most rapidly evolving gene families in plants [32]. The remaining 7536 genes were partially methylated in Col and had reduced methylation to varying degrees in Cvi (clusters 1, 3, and 4; Fig. 1b). The majority of these genes also lacked non-CG methylation in both strains (Fig. 1c), suggesting that the differences in gene body methylation were not due to differential RdDM activity.

To determine whether Cvi gene body methylation patterns were unusual compared to a broader panel of wild-type *Arabidopsis* strains, we performed principal component analysis (PCA) of weighted average CG gene body methylation (calculated as in [33]) for 927 strains characterized by Kawakatsu et al. [19] (Additional file 1: Figure S1). The first principal component explained 92% of the variance in the data, and likely roughly corresponds to overall gene body methylation levels. Cvi was a clear outlier compared to most other strains, suggesting that this degree of gene body hypomethylation is unusual, although not unique, in the global *A. thaliana* population.

### Existing methylation states are stably transmitted for many generations

**Fig. 2:**
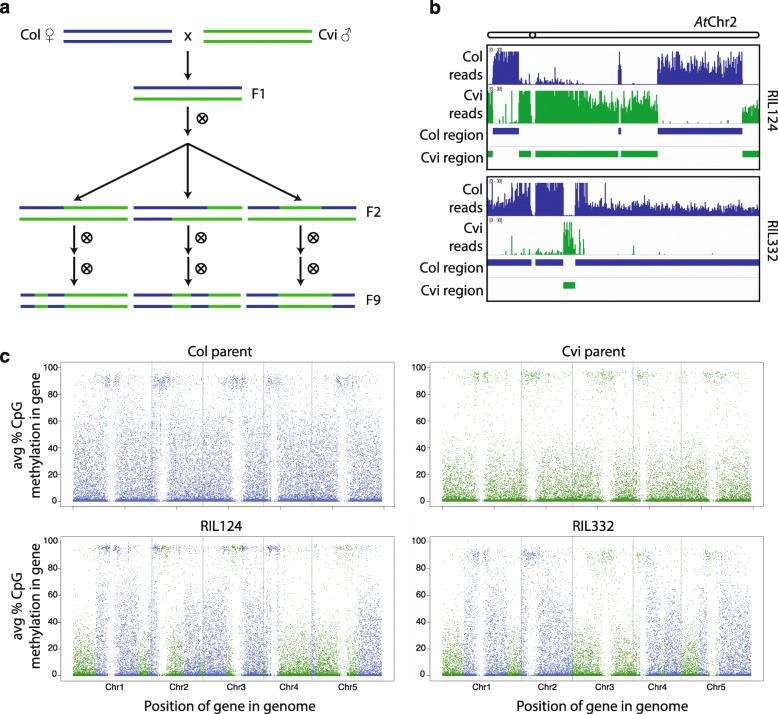
Most gene body methylation is inherited unchanged from the parent-of-origin. **a** The process used by [29] to generate the Col–Cvi RILs. **b** Depth of reads overlapping Col SNPs or Cvi SNPs across chromosome 2 for RILs 124 and 332. Inferred homozygous Col and Cvi regions are shown below the depth tracks. **c** Comparison of gene body CG methylation in Col, Cvi, and two Col–Cvi RILs (first replicate of each sample). *Blue points* represent genes with a Col genotype, *green points* represent genes with a Cvi genotype

Given the striking differences in gene body methylation between Col and Cvi, we evaluated the fidelity with which these different epigenetic states were transmitted to progeny. For these experiments we utilized Col–Cvi RILs, which are homozygous for different combinations of Col- and Cvi-derived sequence in individual RILs (Fig. 2a) [29]. We performed whole-genome bisulfite sequencing on rosette leaves from two biological replicates (siblings) from ten RILs at the F_9_ generation (Additional file 1: Table S1). CG methylation profiles in biological replicates were highly similar, with between-replicate Pearson correlation values of 0.967–0.989 (Additional file 1: Table S2; see “Methods”). We reconstructed the genotype of each RIL at fine scale using reads that overlapped known Col–Cvi SNPs (Fig. 2b; see “Methods”) and determined the set of genes inherited from Cvi and the set inherited from Col in each line. Weighted average CG methylation [33] was calculated across each gene for all samples. In all ten RILs, CG methylation levels in gene bodies were generally stably transmitted according to the underlying genotype: genes inherited from Col remained relatively highly methylated and genes inherited from Cvi remained relatively lowly methylated (Fig. 2c).

### Differences in gene body methylation are not associated with gene expression differences

Because the stable transmission of methylation states led to the preservation of parental methylation levels for individual genes in the RILs, we examined whether differences in gene body methylation within a RIL were associated with differences in gene expression. We performed RNA-seq on leaf tissue from two RILs and from the Col and Cvi wild-type parent lines (Additional file 1: Figure S2; Additional file 1: Table S3). Despite the substantially lower levels of gene body methylation in Cvi, the overall relationship between gene body methylation and gene expression was quite similar between Col and Cvi, with moderately expressed genes associated with the highest levels of gene body methylation in both strains (Additional file 1: Figure S3), as has been previously described [2, 3]. Not surprisingly, PCA demonstrated that the expression of Col genes in the RILs was more similar to the expression of the same genes in the Col parent than in the Cvi parent and vice versa (Additional file 1: Figure S2). Because higher gene body methylation levels are associated with moderately high expression [2, 3, 34], we tested whether body methylated genes were globally more highly expressed in Col than in Cvi. We compared the distribution of FPKM values at Col-inherited genes to Cvi-inherited genes in the RILs (Additional file 1: Figure S3). Because each RIL inherits random sets of genes from each of the parent lines, one set of genes could be inherently more highly expressed than the other simply by chance. To control for this, we also compared the expression of these same sets of genes in each of the parent lines. This analysis was performed over three groups of genes with progressively larger differences in gene body methylation between Col and Cvi (Fig. 1b, clusters 1, 3, and 4). If higher average gene body methylation leads to higher average expression, a shift towards higher expression levels in the Col-derived genes compared to the Cvi-derived genes is expected in the RIL samples, but not in either parent line. However, we found no evidence for such a shift in any of the three groups of genes analyzed (Additional file 1: Figure S3). These data demonstrate that although gene body methylation and expression are correlated, the differences in gene body methylation between Col and Cvi have not led to global changes in expression at body-methylated genes in the RILs (Additional file 1: Figure S3). Our data suggest that, consistent with previous studies [2, 18–20], gene body methylation does not broadly affect gene expression.

### A small number of CG sites consistently fail to maintain the parental methylation state

**Fig. 3:**
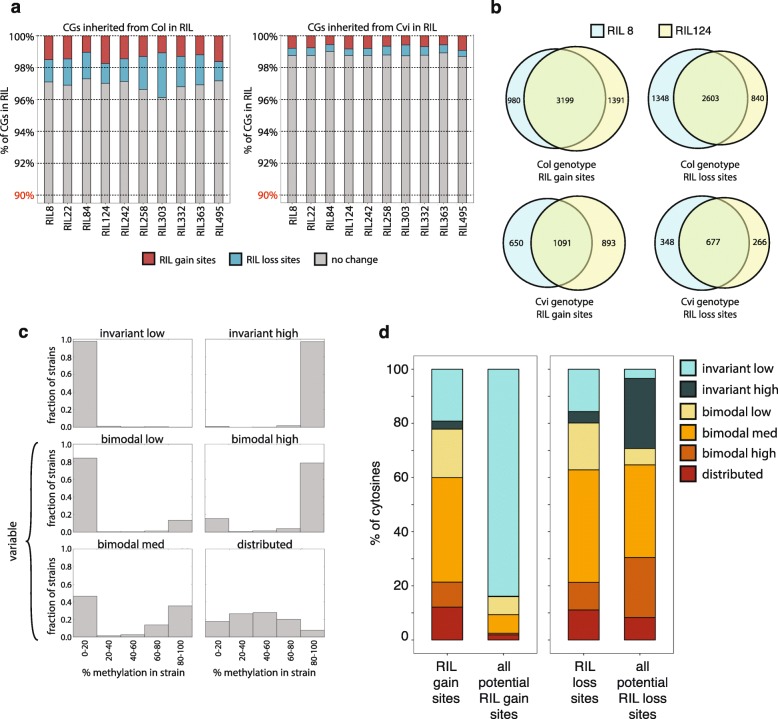
Dynamic genic CGs are rare but shared among RILs. **a** Summary of the fraction of CG sites in genes in each RIL that have gained or lost methylation relative to the parent line, by genotype in the RIL. Note that the *y-axis* begins at 90%. **b** Intersection of dynamic genic cytosines identified in RILs 8 and 124, by genotype. **c** Examples of each of the six classification categories based on methylation across 927 *A. thaliana* strains. Each panel represents data for an individual CG and shows the proportion of strains with methylation levels at that CG in each of the five bins indicated. **d** Percentage of cytosines classified into each of the six categories shown in **c** for different groups of CGs from Col-derived regions in RIL 8. The *left panel* compares distribution at CG sites where the RIL has gained methylation relative to the parent line (RIL gain sites) to CG sites where the parent line had sufficiently low methylation to enable gain of methylation in the RIL (all potential RIL gain sites). The *right panel* compares CG sites where the RIL has lost methylation (RIL loss sites) to CG sites where the parent line had sufficient methylation to be lost (all potential RIL loss sites)

Although the methylation state of individual genes was highly conserved by genotype in the RILs (Fig. 2), data from two biological replicates per line allowed us to identify with high confidence between 10,000 to 20,000 “dynamic” genic CGs in each RIL that either gained or lost methylation relative to the parent line. Dynamic sites corresponded to between 1 and 4% of all genic CGs (Fig. 3a; see “Methods”). In Col-derived genes, roughly equal numbers of CGs gained or lost methylation; the same was true in Cvi-derived genes, although these genes contained fewer dynamic CGs in total (Fig. 3a). Most dynamic CGs were in genes lacking non-CG methylation in the parent (Additional file 1: Figure S4). In contrast to genes, and as demonstrated in prior studies [35, 36], methylation in TEs was generally more stable than in genes, with only 0.6–1.7% of CG sites differentially methylated between the RIL and parent line (Additional file 1: Figure S5). We validated four loci containing at least one dynamic cytosine using locus-specific bisulfite PCR (Additional file 1: Figure S6), after first confirming by DNA sequencing that the putative dynamic CGs did not correspond to unannotated SNPs. All four loci were validated, behaving exactly as indicated from the whole-genome bisulfite sequencing data. Using the dynamic CGs, we calculated the ratio of the rate of methylation loss to the rate of methylation gain for each RIL (Additional file 1: Figure S7; see “Methods”). TEs had much lower ratios of methylation loss to methylation gain than did genes, regardless of parental genotype, consistent with their much higher CG methylation levels. For genic CGs, the ratio of loss to gain was higher in the Cvi-derived regions than in the Col-derived regions for most RILs. These findings are consistent with the lower gene body methylation levels found in Cvi-derived regions.

We also examined the effect of these dynamic CGs on gene expression. We found that genes with more dynamic cytosines had similar expression in the RIL compared to the parent line, regardless of whether those dynamic cytosines represented gain or loss of methylation in the RIL (Additional file 1: Figure S3). These results again suggest that alterations in gene body methylation do not alter gene expression.

### Changes in CG methylation are not stochastic

Although each RIL contained only a small fraction of dynamic cytosines, the same CG sites were often identified as dynamic in multiple RILs, at a much higher rate than would be expected by chance (Fig. 3b; hypergeometric test *p* ≈ 0 for all four panels). This was true for any pair of RILs, for both sites that gained methylation in the RIL not present in the parent line (RIL gain sites) and sites that lost methylation compared to the parent (RIL loss sites). These data suggest that some genic CGs are consistently more prone to methylation changes than others, in agreement with similar findings from other studies [35–37]. Dynamic CGs also tended to occur at sites where the Col parent line was more methylated than Cvi (Additional file 1: Figure S8; one-sided hypergeometric test *p* ≈ 0 for both panels in Figure S8a), and to a lesser extent at sites where the Col parent line was less methylated than Cvi (Additional file 1: Figure S8; hypergeometric test *p* ≈ 0 for the left panel and *p* = 1.7 × 10^–67^ for the right panel in Figure S8b). Thus, dynamic cytosines are predominantly a subset of sites where the Col and Cvi parent lines are already differentially methylated.

To determine whether there was evidence for dynamic methylation at these same sites outside of the Col-Cvi RIL context, we examined how variable methylation was at these sites within the natural *A. thaliana* population. Each genic CG (n = 1,634,516) in the genome was classified into one of six categories according to its methylation variability among 927 wild-type strains [19] (Fig. 3c; see “Methods”). “Invariant low” and “invariant high” classifiers designated CG sites with consistently low or high levels of methylation across the 927 strains, respectively, whereas the remaining four categories indicated variable methylation levels of differing types (Fig. 3c). Dynamic CGs that gained methylation in the RILs were more likely to be classified into the four “variable” categories compared to all CGs that could have gained methylation (Fig. 3d). A similar, although less strongly biased, relationship was also observed for dynamic CGs that lost methylation in the RILs compared to all CGs that could have lost methylation. Overall, these results indicate that CGs with variable methylation levels among different wild-type strains were more likely to be dynamic CGs in the RILs. This suggests that the dynamic nature of these CGs is not specific to the Col–Cvi RILs, but is instead an inherent property of particular CG sites in the *A. thaliana* genome, regardless of strain background.

### Dynamic CGs are clustered and share local methylation features

Because our data indicated that dynamic genic CGs in the RILs were shared (Fig. 3b), we looked for features that could distinguish these sites from non-dynamic genic CGs. Specifically, we sought features that could distinguish RIL gain sites from other genic CGs with low methylation in the parent lines (potential RIL gain sites) and/or could distinguish RIL loss sites from other highly methylated genic CGs in the parent lines (potential RIL loss sites). (Only lowly methylated sites in the parent lines have the potential to gain methylation in the RILs and vice versa*.*) We evaluated whether DNA sequence, methylation, or small RNA features were associated with each type of dynamic CG.

**Fig. 4:**
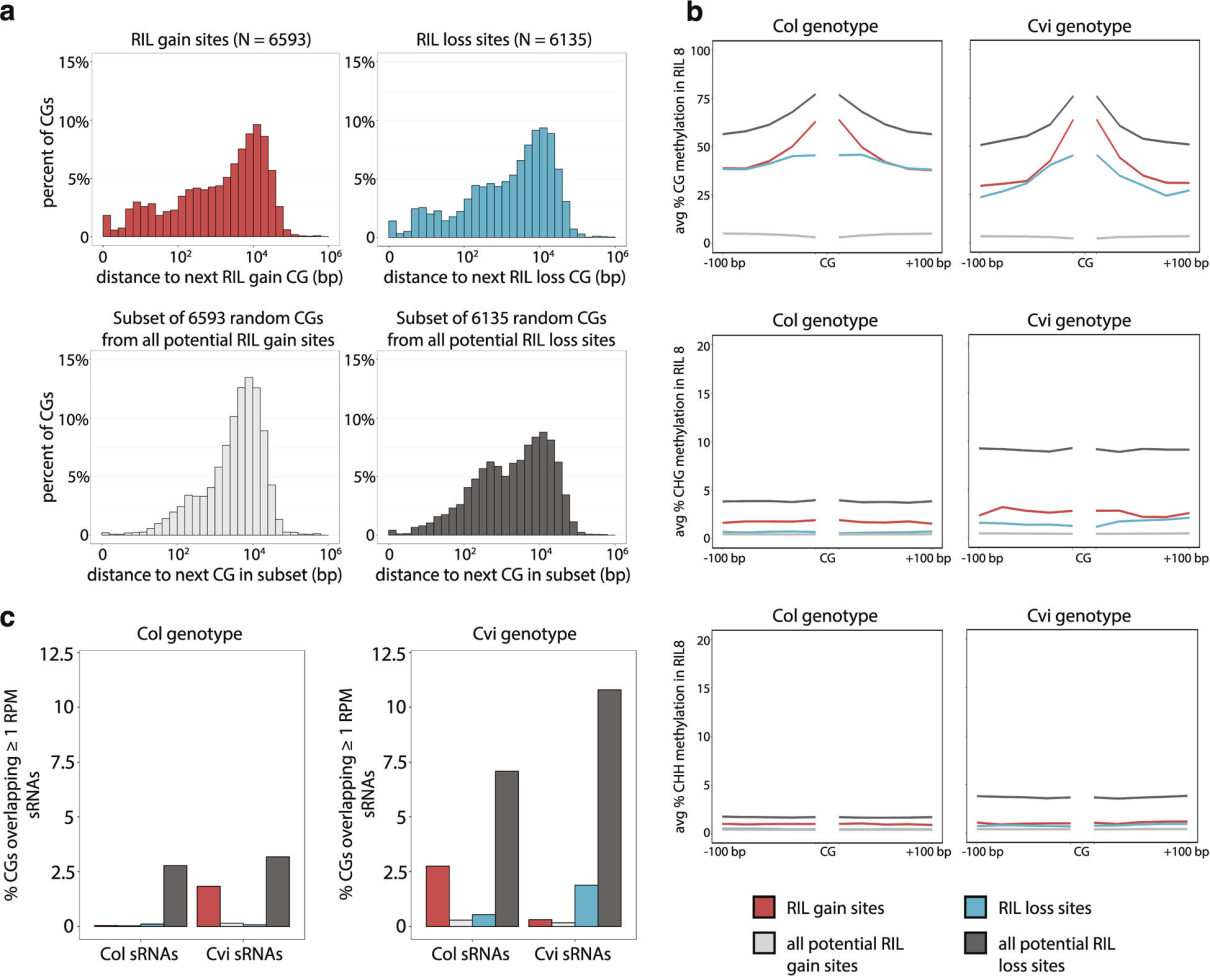
Physical clustering, local methylation, and small RNA (sRNA) features associated with dynamic cytosines. **a** Distance to the next closest cytosine in specified groups of CGs. Data shown for CGs in RIL 8 inherited from Col. A small number of distances >10^6^ bp were omitted from the plots. **b** Average CG (*top*), CHG (*middle*), and CHH (*bottom*) methylation profiles in RIL 8 in the 200 bp surrounding indicated CGs. **c** Percentage of indicated genic CGs from Col-derived (*left*) and Cvi-derived (*right*) regions in the RILs overlapping at least 1 RPM of 24-nucleotide sRNAs from Col or Cvi embryos. Legend same as **b**

RIL gain sites were physically much closer to each other than random subsets of equal size drawn from all CGs with low methylation levels in the parent line (Fig. 4a; z-score = 49.3, *p* ≈ 0; see “Methods”), suggesting that gain of methylation in the RILs occurred at discrete loci. RIL loss sites were also significantly closer to each other than a random number of sites highly methylated in the parent, but to a much lesser extent (Fig. 4a; z-score = 13.25, *p* ≈ 0; see “Methods”). In addition, dynamic CG sites were not randomly distributed within gene bodies. RIL gain sites were strongly depleted at the 5′ end of genes relative to CGs randomly drawn from the set of all potential RIL gain sites (Additional file 1: Figure S4; see “Methods”). RIL loss sites were also depleted near the transcription start site and transcription termination site, but enriched in the 3′ portion of genes. Interestingly, while RIL loss sites were evenly distributed around intron–exon boundaries, RIL gain sites were enriched at those boundaries and in introns, but somewhat depleted in exons (Additional file 1: Figure S4; see “Methods”). These differences suggest that RIL gain and RIL loss events may occur through different mechanisms and might, therefore, be associated with different features.

We examined whether dynamic cytosines were associated with specific local methylation patterns in the 200 bp flanking each site. RIL gain sites occurred in regions with intermediate levels of local CG methylation; proximal methylation (red line in Fig. 4b) was higher than that observed around genic CGs that lack methylation (light gray line in Fig. 4b), but lower than that around genic CG sites that are highly methylated in the parent (dark gray line in Fig. 4b). Similarly, RIL loss sites (blue line in Fig. 4b) also occurred in regions with intermediate methylation. Dynamic CGs, particularly RIL gain sites, were associated with low levels of non-CG methylation, although to a lesser extent than genic sites already methylated in the parent lines (Fig. 4b). De novo methylation through the RdDM pathway could be one mechanism to explain gain of methylation in the RILs, although gene body methylation is generally not associated with small RNAs (sRNAs) [2]. We compared the levels of 24-nucleotide sRNAs from Col leaves (Additional file 1: Figure S9) [38] and Col and Cvi embryos (Fig. 4c) around dynamic CGs. Less than 3% of RIL gain sites from either parent were associated with sRNAs, suggesting that RdDM activity does not explain methylation gain at the majority of these sites. Interestingly, of these 3%, the Col-derived RIL gain sites were specifically enriched for sRNAs only found in Cvi, whereas the Cvi-derived RIL gain sites were enriched for sRNAs from Col (Fig. 4c; Additional file 1: Figure S9). These data suggest that gain of methylation initiated by RdDM occurred in *trans* at these sites, likely in the ColxCvi F_1_ plant.

We also examined local sequence composition around dynamic cytosines. We found that RIL loss sites did not have any substantial differences in C context or GC content compared to methylated sites in the parent line (Additional file 1: Figure S10). RIL gain sites, however, were in regions that were locally somewhat GC-poor and depleted of CG sites compared to all sites unmethylated in the parent line. Using DREME [39], we identified sequence motifs enriched in the 200 bp around RIL gain sites compared to background (Additional file 1: Figure S10; see “Methods”), including TGCWR and RCATW. However, all of the sequence features associated with RIL gain sites (CG depletion, reduced GC content, and identified sequence motifs) were also found around CGs stably methylated in the parent lines and the RILs, as well as around sites that were methylated in the parents but lost methylation in the RILs (Additional file 1: Figure S10), suggesting that they are more general features of methylated CGs in gene bodies. Thus, RIL gain sites are most likely to arise in places with local sequence features resembling those around methylated DNA, even though the parent line is actually unmethylated at these sites. RIL loss sites, which by definition must occur at parentally methylated CGs, are not distinguishable from other parentally methylated sites based on the local sequence features examined here.

### Prediction of dynamic genic CGs using a logistic regression framework

**Fig. 5:**
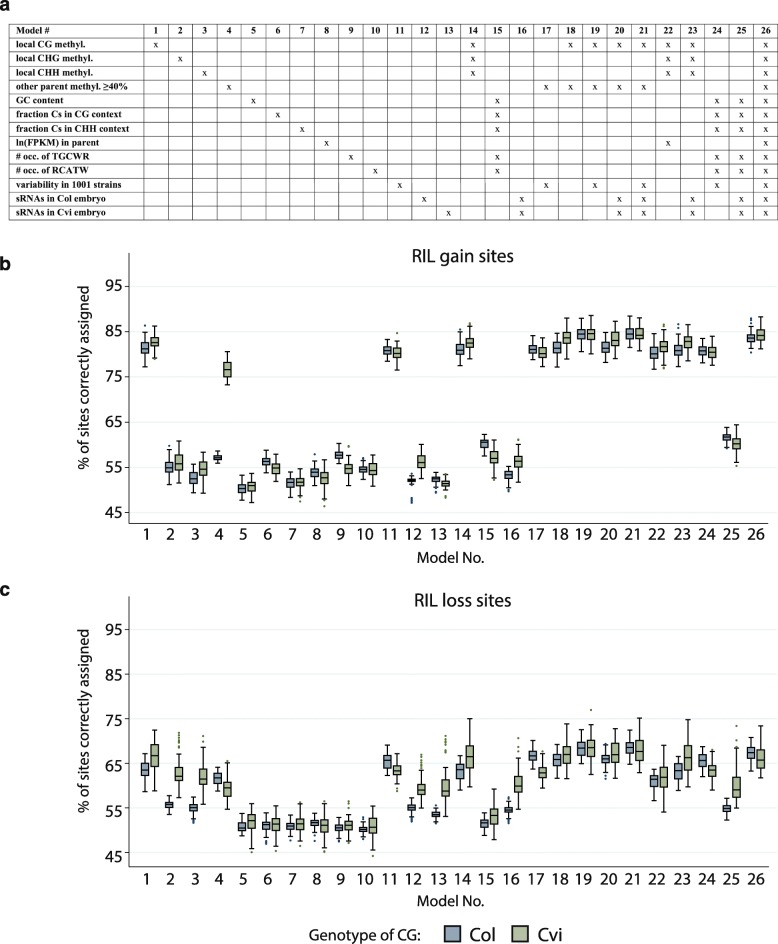
Prediction of dynamic cytosines by 26 different logistic regression models. **a** Combination of predictors used in each of the 26 models tested. **b**, **c** Distribution of percentage of sites correctly assigned when using the indicated model to predict RIL gain sites (**b**) or RIL loss sites (**c**) in the RILs. Each boxplot represents 100 points—ten predictions based on different randomly drawn background values (see “Methods”) for each of the ten RILs. Accuracy of 50% is no better than random

To assess how informative the various features associated with dynamic CGs are in determining where dynamic CG sites occur, we used a logistic regression framework [40] to test 26 models consisting of various combinations of 13 features, including local DNA methylation level, sequence composition, presence of sequence motifs, presence of sRNAs, gene expression level, and population variability (Fig. 5a). We assessed the ability of each model to correctly identify RIL gain sites, RIL loss sites, and non-dynamic sites in a subset of the data after training the model on a different subset (see “Methods”). Subsets were selected to contain 50% RIL gain or loss sites and 50% sites from the appropriate background. Thus, prediction accuracy above 50% indicated that a model performed better than by chance.

Models incorporating either local CG methylation levels (model 1) or methylation variability across the 927 *A. thaliana* strains (model 11) performed best at predicting RIL gain sites (Fig. 5b). Alone, each model correctly predicted gain sites in either Col- or Cvi-derived regions ~82% of the time, much higher than the ~50% accuracy achieved by randomly guessing. However, models incorporating both predictors simultaneously performed only slightly better, suggesting that these two features contained more or less the same information with respect to predicting RIL gain sites. Similarly, the complete model (model 26) performed barely better than either of these two predictors individually, suggesting that the other predictors contribute very little, if any, useful information in the estimation of CG gene body methylation gain. In general, models performed equally well for gain sites in Col- and Cvi- derived regions in the RILs. An exception was model 4, which encoded whether the parent from which the CG was not derived had methylation at that CG. Knowing the methylation state in Col strongly informed which sites gained methylation in Cvi-derived regions in the RILs, consistent with other observations (Additional file 1: Figure S8). Models 1 and 11 also generally performed the best for predicting RIL loss sites (Fig. 5c). Interestingly, several regression models that used non-CG methylation (models 2 and 3) and sRNAs (models 12 and 13) performed better in Cvi-derived regions than in Col-derived regions at predicting RIL loss sites, but did not predict RIL gain sites for either genotype. These results suggest that RdDM influences RIL loss events only in Cvi-derived regions. Overall, however, all models were considerably less able to accurately distinguish RIL loss sites than RIL gain sites. In conclusion, although there likely remain features not examined here that are associated with dynamic cytosines, particularly RIL loss sites, our models achieve substantially higher prediction accuracy than random.

To extend our findings on dynamic CG gene body methylation beyond the specific Col–Cvi RIL context, we used our regression approach to determine whether genic CGs variably methylated across *A. thaliana* strains could be distinguished from a background of invariably methylated CGs (methylated or unmethylated) using only local methylation levels as predictors (Additional file 1: Figure S11). All seven models tested performed better than random. Local CG methylation was the strongest predictor for which sites were variably methylated compared to unmethylated CGs (conceptually similar to RIL gain sites). However, in comparing variably methylated sites to methylated sites (conceptually similar to RIL loss sites) local CG methylation was not as strong a predictor. This is consistent with our finding that RIL loss sites are less well predicted than RIL gain sites (Fig. 5). We next examined whether these relationships existed in two other species with distinct genic methylation profiles. *Zea mays* (maize) has extensive CG methylation in gene bodies, but unlike *A. thaliana* also has high levels of genic CHG methylation (Additional file 1: Figure S11) [41]. *B. distachyon* has higher genic CG methylation than *A. thaliana* but lower genic CHG methylation than maize (Additional file 1: Figure S11) [42]. We used bisulfite sequencing data from five maize inbred lines [43] and seven *B. distachyon* inbred lines [42] to identify CGs that were variably methylated across strains within each species (see “Methods”). CG and non-CG methylation levels around variably methylated sites were intermediate compared to other CGs in both maize and *B. distachyon* (Additional file 1: Figure S11), which is similar to our observations in *A. thaliana* (Fig. 4b). We then repeated our logistic regression analysis on these species using the seven models that incorporate local methylation as predictors (Additional file 1: Figure S11). As in *A. thaliana*, levels of surrounding CG methylation strongly predicted which sites were variably methylated in maize and in *B. distachyon*, particularly in comparison to invariably unmethylated CGs. Consistent with our previous results, little additive effect was observed in any model combining multiple predictors, suggesting that the different types of methylation do not behave independently with respect to predicting variably methylated sites. Thus, despite differences in gene body methylation patterns between *A. thaliana*, maize, and *B. distachyon*, the overall relationship between variably methylated CGs and local methylation is similar, indicating that our results on dynamic genic CG methylation are likely not specific to *A. thaliana*, but are instead broadly applicable.

## Discussion

Recent studies have highlighted the natural epigenetic variation present within the *A. thaliana* population [12, 19]. Here we assessed the fidelity of methylation inheritance, and uncovered features associated with non-random changes in gene body methylation, by taking advantage of a RIL population created from two wild-type strains with large, naturally occurring differences in this type of methylation. Our results demonstrate that gene body methylation levels are generally stably inherited, with individual genic methylation levels in the RILs resembling those in the parent genotype even after nine generations of separation. A methylation analysis of soybean RILs also found that methylation was generally inherited according to genotype [18], but did not assess heritability at specific genic CG sites. Several other studies have suggested that CG methylation states can be quite stable [14, 23, 24]. However, examination of methylation across multiple generations in lines propagated by single-seed descent from Col (mutation accumulation lines or MA lines) concluded that CGs in gene bodies had higher epimutation rates compared to CGs in TEs or other regions [35, 36, 44], suggesting that gene body methylation is one of the least stable types of methylation in the genome. In agreement with previous results from the MA lines, the dynamic CGs identified in this study were more common in gene bodies than in TEs [35, 36, 44]. How, then, are gene body methylation patterns conserved on evolutionary time scales? Gene body methylation levels, at equilibrium, are determined by the ratio of the rate of methylation loss to the rate of methylation gain. Cvi-inherited genic CGs had a higher ratio of loss to gain in most RILs than did Col-inherited genic CGs, consistent with the lower gene body methylation level observed in the Cvi-inherited regions (Additional file 1: Figure S7). Additionally, similar numbers of CGs gained methylation and lost methylation in Cvi-derived regions in each RIL, and the same was true in Col-derived regions (Fig. 3a). These observations suggest that gene body methylation levels in the RILs are already at equilibrium, with a different equilibrium methylation level for Col- and Cvi-derived genes. If this is the case, Col- and Cvi-derived genes are likely to retain the parental methylation state over very long time periods, rather than move slowly towards a common methylation level. This suggests that although epimutation rates may be highest for CGs in gene bodies [44] (Additional file 1: Figure S7), equal flux in both directions (Fig. 3a) will lead to overall methylation levels remaining consistent over time.

Genic methylation in Cvi-derived CGs in the RILs is, somewhat counter-intuitively, more stable than in Col. Both gain and loss of methylation were substantially less frequent at Cvi-derived CGs than at Col-derived CGs (Fig. 3a). This observation is seemingly contradictory because it is Cvi, not Col, that has unusual gene body methylation compared to the rest of the *A. thaliana* population (Additional file 1: Figure S1). One possible explanation is that because Cvi has already lost methylation at many genic CG sites, the methylated CGs that remain are those that are particularly stable and potentially reinforced by other mechanisms. Consistent with this hypothesis, methylated genic CGs in Cvi are much more likely to be associated with local non-CG methylation (dark gray line in Fig. 4b) and sRNAs (Fig. 4c) than methylated CGs in Col. This suggests that a greater proportion of genic CG methylation in Cvi is reinforced by RdDM than in Col.

Gain of methylation in the RILs did not appear to involve RdDM activity at most CGs; nearly all RIL gain sites were not associated with sRNAs from either Col or Cvi (Fig. 4c), and sRNA levels poorly predicted RIL gain sites (Fig. 5b). However, because we did not profile sRNAs in the RILs themselves, we cannot definitively rule out the possibility that RIL gain sites may arise in these lines due to the action of sRNAs not observed in parental embryos or leaves. The small number of RIL gain sites that were associated with sRNAs contained almost exclusively sRNAs specific to the parental genotype from which that CG was not inherited (Fig. 4c). These are likely strain-specific RdDM targets that became methylated in the ColxCvi F_1_ plants through *trans*-acting sRNAs. This phenomenon has been previously observed [45], though more often in TEs than in gene bodies. The association of embryo and leaf sRNAs with gain of methylated CGs in gene bodies, although limited, supports the hypothesis that gene body methylation can be acquired through an RdDM mechanism, with non-CG methylation lost when reinforcing sRNAs are no longer present [3, 46].

RIL gain sites were highly predictable because they possessed features that were easily distinguishable from stably unmethylated DNA. Intermediate levels of proximal CG methylation and higher methylation variability within the *A. thaliana* population were strongly associated with RIL gain sites. By contrast, RIL loss sites were less predictable because they shared many features with stably methylated sites and were thus difficult to distinguish from these sites. However, the best models could predict both gain and loss sites much better than random. Interestingly, different combinations of predictors in the models rarely had any additive effect on the ability to predict RIL gain and loss sites: if either local CG methylation or methylation variability are known, the remaining predictors are largely dispensable. This suggests that many of these predictors are either correlated or otherwise contain similar information relevant to predicting dynamic cytosines. This would not be surprising in several cases; the presence of sRNAs would be expected to correlate with local CHH methylation, for example. However, the lack of additivity in some models can reveal additional information about the predictors. For example, both gain of methylation in Cvi-derived regions and loss of methylation in Col-derived regions were more likely at sites where Col is methylated but Cvi is not (Additional file 1: Figure S8). This led to increased prediction accuracy for RIL gain sites in Cvi-derived regions and for RIL loss sites in Col-derived regions when the methylation state of the other parent was known (Fig. 5, model 4). Methylation changes could be more likely at these sites because they are more likely to be variably methylated across strains in general, which would explain why a model combining both of these predictors (Fig. 5, model 17) does not perform better than either predictor alone (Fig. 5, models 4 and 11). By contrast, CGs where Cvi is methylated but Col is not are concentrated in a small number of genes and are likely Cvi-specific RdDM targets (Fig. 1b, c). Our results suggest that RdDM plays a limited role in gain or loss of methylation in the RILs, which likely accounts for both the lower overlap between these sites and dynamic CGs (Additional file 1: Figure S8) and for the lack of predictive power for Col-derived RIL gain sites and Cvi-derived RIL loss sites using model 4. Overall, the regression results further suggest that dynamic cytosines do not occur at random and demonstrate that some features associated with dynamic sites are strongly predictive, whereas others are only weakly associated.

The tendency of genic CGs in regions of intermediate local methylation to be less stably methylated is not limited to the *A. thaliana* RILs used in this study. Local methylation levels predict methylation variability to a similar extent in maize and *Brachypodium* as in *A. thaliana*, despite the divergent gene body methylation profiles found among these three species. Thus, these results are not specific to a certain type of genome or genotype, but reflect more generally on fundamental properties of gene body DNA methylation stability.

## Conclusions

We have provided a detailed view of how gene body methylation is inherited in *A. thaliana* mosaic genomes. Our results demonstrate that two different equilibrium gene body methylation levels can be independently maintained over many generations in a RIL. We also show that genic CG sites that become differentially methylated compared to the parent are conserved and predictable, and appear to belong to a larger group of CG sites that are highly variable across the *A. thaliana* population.

## Methods

### Plant material

Col–Cvi RILs and their parent lines were obtained from the lab of Fred Ausubel (originally obtained from INRA Versailles, generated by [29]). Plants were grown in a greenhouse in soil with 16 h light at 21 **°**C.

### Bisulfite sequencing

Approximately 100 mg of leaf tissue was harvested from two individual 3-week-old rosettes for ten RILs (lines 8, 22, 84, 124, 242, 258, 303, 332, 363, and 495) and from the Col and Cvi parent lines. Tissue was pulverized with a Qiagen TissueLyser II, and DNA was extracted using the Qiagen DNeasy plant mini kit (catalog number 69104). DNA was bisulfite converted using the MethylCode bisulfite conversion kit (Invitrogen, catalog number MECOV-50). Bisulfite sequencing libraries were constructed using the EpiGnome Methyl-seq kit from Epicentre (now the TruSeq DNA methylation kit from Illumina, catalog number EGMK81312, index primers provided separately with catalog number EGIDX81312). Reads were sequenced on an Illumina HiSeq2000 using a 40 × 40, 50 × 50 or 100 × 100 bp paired-end protocol at the Whitehead Institute Genome Technology Core (Additional file 1: Table S1). Reads were quality filtered using trim_galore v.0.3.7 [47] with parameters --phred64 --paired -a AGATCGGAAGAGCACACGTCTGAAC -a2 AGATCGGAAGAGCGTCGTGTAGGGA --stringency 3 -r1 32 -r2 32 --clip_R1 8 --clip_R2 8 -q 25 and all other parameters default. Filtered reads were aligned to the genome using Bismark v0.16.1 [48] with mapping parameters -q --bowtie1 --phred64-quals -n 1 -l 40 -k 2 --best --minins 0 --maxins 500 and all other parameters default. To improve the mapping of Cvi-derived reads, reads for all samples were initially mapped to a Col–Cvi metagenome, which consisted of the Cvi pseudogenome, created by substituting the Cvi allele of all Col/Cvi SNPs into the TAIR10 assembly, appended to the TAIR10 (Col) sequence. Reads mapping ambiguously to the metagenome were then remapped to TAIR10 using Bismark, with the same parameters noted above. PCR duplicates were removed with a script provided with the Bismark installation [48], which avoids introducing bias at this step by choosing a random read to keep from each set of presumed PCR duplicates. All reads were then classified based on overlapping SNPs into reads from Col, reads from Cvi, and all other reads using a custom script (assign_to_allele.py; see “Availability of data and materials” section below). The Bismark methylation extractor function was used to obtain methylation data from all mapped reads.

### Determining RIL genotype

Coverage of Col- and Cvi-derived reads was obtained over non-overlapping 200-bp windows using the bedtools coverage function. Depth values for each window were smoothed using the moving average of a sliding window of 51 windows, centered on the window being smoothed. Preliminary genotype determinations were made by considering all windows with at least 2 depth in both strains combined (after smoothing), and assigning windows with [Col depth] > 1.5 × [Cvi depth] and [Col depth] − [Cvi depth] > 2 to Col, and windows with [Col depth] × 1.5 < [Cvi depth] and [Cvi depth] − [Col depth] > 2 to Cvi. Regions with abs([Col depth] − [Cvi depth]) < 2 but with [Col depth] + [Cvi depth] > 2 were called heterozygous, while all other windows were considered undetermined. Adjacent windows with the same genotype call were merged to obtain the initial set of homozygous Col or homozygous Cvi regions. These initial regions were refined by iteratively merging small “undetermined” windows into bigger flanking regions. Briefly, if a small region (<2000 bp) was flanked on both sides by larger regions with the same assignment (e.g., both are “homozygous Col”), then the small region was given the same assignment. This was repeated until genotype assignments did not change. Code for this analysis is provided in script call_regions.R (see “Availability of data and materials” section). The script was run with parameters --mindepth 2 --strain1 “Col” --strain2 “Cvi” and all other parameters default.

### Identifying differentially methylated cytosines

CG methylation is typically similar for the cytosines on opposite strands because of the way CG methylation is maintained. Thus, treating the two symmetrical cytosines in CG dinucleotides as independent cytosines is not usually appropriate. Therefore, we identified all CGs for which there were data on both strands, and used a two-sided Fisher’s exact test to test if there was a significant difference in methylation at symmetrical sites. CGs with a corrected *p* value <0.05 and a difference in methylation greater than 40% between the two strands were considered inconsistent and were censored from all remaining analyses; this occurred at less than 0.2% of all cytosines with data on both strands. All other CGs with data on both strands were assigned an overall methylation score equal to the weighted mean of methylation on the forward and reverse strands, and were treated as a single record for all subsequent analyses. The script for this process is get_CG_consistency.sh (see “Availability of data and materials” section). Additionally, all cytosines overlapping a known Col/Cvi SNP were censored to avoid errors in methylation calls. To identify differentially methylated cytosines between two samples (e.g., Col versus Cvi), we compared the number of methylated/unmethylated reads in sample 1 to sample 2 and performed a two-sided Fisher’s exact test. Only cytosines with at least 5 read coverage in all sample comparisons were used, and the test was conducted separately between all four possible combinations of replicates (e.g., Col 1 versus Cvi 1, Col 2 versus Cvi 1, Col 1 versus Cvi 2, Col 2 versus Cvi 2). *P* values for each pairwise comparison were corrected for multiple testing using the Benjamini–Hochberg method. Cytosines with a corrected *p* value below 0.05 and a difference in percentage methylation greater than 40, 40, or 20% (for CGs, CHGs, and CHHs, respectively) were considered significantly differently methylated and assigned a “significance score” equal to 1 if sample 1 was more methylated than sample 2, or −1 if sample 1 was less methylated. Cytosines not significantly different were assigned a score of 0. Once this was performed for all four pairwise comparisons separately, an overall significance score was calculated by summing together the four separate significance scores, resulting in scores in the range of [−4,4]. Cytosines with an overall score ≥3 were considered significantly more methylated in sample 1 than sample 2, while scores ≤ −3 were considered significantly more methylated in sample 2 than sample 1.

### Calculating correlation between replicates

Using the corrected CG methylation data for each sample, we calculated the Pearson correlation between all pairs of samples using Stata’s pwcorr command. All CGs with non-missing data in both samples were used to evaluate correlation between a given pair of samples.

### Estimating rate of gain and loss of genic methylation in the RILs

Because we required a minimum difference in methylation of 40% in order to identify a CG as differentially methylated in the RIL compared to the parent line, we considered all CGs with ≥40% methylation in the parent line as potential RIL loss sites and all CGs with ≤60% methylation as potential RIL gain sites. We then estimated the rate of gain and loss of methylation for each RIL as:

$$ \mathrm{Rate}\ \mathrm{of}\ \mathrm{gain} = \left[\ \mathrm{Number}\ \mathrm{of}\ \mathrm{observed}\ \mathrm{RIL}\ \mathrm{gain}\ \mathrm{sites}\ \right]\ /\ \left[\ \mathrm{Number}\ \mathrm{of}\kern0.5em \mathrm{potential}\ \mathrm{RIL}\ \mathrm{gain}\ \mathrm{sites}\right] \ast 100 $$

$$ \mathrm{Rate}\ \mathrm{of}\ \mathrm{loss} = \left[\ \mathrm{Number}\ \mathrm{of}\ \mathrm{observed}\ \mathrm{RIL}\ \mathrm{loss}\ \mathrm{sites}\ \right]\ /\ \left[\ \mathrm{Number}\ \mathrm{of}\;\mathrm{potential}\ \mathrm{RIL}\ \mathrm{loss}\ \mathrm{sites}\right] \ast 100 $$

This was calculated separately for Col-derived and Cvi-derived CGs. The ratio of the rate of loss to gain was then calculated as:

$$ \mathrm{Ratio} = \left[\ \mathrm{Rate}\ \mathrm{of}\ \mathrm{loss}\ \right]\ /\ \left[\ \mathrm{Rate}\ \mathrm{of}\ \mathrm{gain}\ \right] $$

### PCA of gene body methylation levels in 927 strains

We obtained weighted average CG methylation levels in 927 *A. thaliana* strains [19], considering only positions with at least 5 read coverage in the calculation (after processing data at symmetrical CGs as described in “Identifying differentially methylated cytosines”). If weighted average methylation levels were calculated over fewer than five CG sites (with ≥5 coverage each), that observation was censored. We dropped all genes with censored or missing methylation values in at least one of the 927 strains, retaining 14,736 genes with data in all strains. PCA was performed using the R function prcomp, and the projection of each strain onto the first two principal components is plotted in Additional file 1: Figure S1.

### Classifying CGs according to methylation variability across 927 *A. thaliana* strains

Using CG methylation data from 927 *A. thaliana* strains [19], we classified CGs into a number of categories based on the variability of methylation levels across these strains (Additional file 1: Figure S12). Briefly, CGs covered by at least five reads in at least 627 of the strains (after processing data at symmetrical CGs as described in “Identifying differentially methylated cytosines”) were used for this analysis. Data for all strains with at least 5 read coverage at that CG were binned into five equal bins according to methylation level (0–20, 20–40%, etc.). The distribution of the strains among these bins, a vector of length 5 summing to 1, was used for classification. All bins corresponding to local maxima (peaks) were identified, with peaks required to contain at least 5% of strains. CGs were classified into various categories based on the number of peaks in the distribution and how much of the density of the distribution was in those peaks (Additional file 1: Figure S12). Subcategories were used to indicate where the majority of the density of the distribution resided. For unimodal distributions, the subcategory was the peak location itself (Additional file 1: Figure S12; the five bins from lowest methylation to highest are named “lo”, “medlo”, “med”, “medhi”, and “hi”). For bimodal distributions, the subcategory was “mostly” if the highest peak was more than four times the second peak (e.g., “mostly lo”), “biased” if the highest peak was more than 1.5× the second peak (e.g., “biased hi”), and “similar” otherwise. Trimodal distributions were not assigned subcategories. We then grouped these categories into six overall classes (Fig. 3). All CGs classified as “unimodal sharp” or “unimodal inter” (Additional file 1: Figure S12) were grouped into the “invariant” class, with subcategories “lo” or “medlo” considered “invariant low” and subcategories “hi” or “medhi” considered “invariant high”. All CGs classified as “bimodal sharp” or “bimodal inter” were grouped into the “bimodal” class, with subcategories “mostly hi” and “mostly medhi” considered “bimodal high”, subcategories “mostly lo” and “mostly medlo” considered “bimodal low”, and all “biased” or “similar” subcategories considered “bimodal med”. All other categories were grouped into the “distributed” class. The Python script used to perform the classification is classify_variation_across_samples.py (see “Availability of data and materials” section).

### Physical clustering of RIL gain or RIL loss sites

We determined whether the distribution of distances between RIL gain sites or RIL loss sites shown in Fig. 4a represented a significant deviation from the expected distribution. To simplify comparisons between histograms, we used the fraction of distances ≤100 bp (f_100_) as a measure of how strongly a particular group of CGs was clustered. Since RIL gain and loss sites are a subset of a larger population of CG sites (the set of all potential RIL gain or loss sites), we obtained the background distribution of f_100_ by repeatedly drawing random subsets, of size equal to the number of true RIL gain or true RIL loss sites, from the set of all potential RIL gain or RIL loss sites. This was repeated N = 1000 times, and the mean and standard deviation of f_100_ across the random samples was then used to calculate the z-score and *p* value corresponding to the f_100_ observed using the true RIL gain or loss sites. Results are shown in Table 1.

**Table 1:** Data for physical clustering of dynamic cytosines

	Background mean f_100_	Background f_100_ s.d.	Observed true value of f_100_	Corresponding z-score	*P* value
RIL gain CGs	0.06	0.0029	0.203	49.31	0
RIL loss CGs	0.116	0.004	0.169	13.25	0

*s.d.* standard deviation

### Distribution of RIL gain or loss sites across gene bodies and intron–exon boundaries

To determine how RIL gain and RIL loss sites are distributed around gene bodies and intron–exon boundaries, we generated metaplots over these features of the average fraction of all potential RIL gain or loss sites that are true RIL gain or loss sites (see “Availability of data and materials” section, script ends_analysis.sh, and “Methylation profile plots” section below). The value plotted does not show the actual distribution of RIL gain or loss sites, but rather their distribution relative to the set of all potential RIL gain or loss sites. Therefore, if the true RIL gain or loss sites represent random draws from the set of all potential RIL gain or loss sites, without regard to position within genes or around intron–exon boundaries, the expected distribution should be roughly uniform across these features, as confirmed in Additional file 1: Figure S4, where an equal number of CGs was drawn randomly from the set of all potential RIL gain or loss sites for comparison (see gray lines in each plot).

### Methylation profile plots

The script used to generate methylation profile plots like those in Fig. 4b is ends_analysis.sh (see “Availability of data and materials” section). For plots in Fig. 4b, parameters used were -I 0 -O 100 -w 20. For plots in Additional file 1: Figure S4, parameters used were -I 500 -O 0 -w 20 for part B (feature = genes) and -I 200 -O 200 -w 20 for part C (feature = exons). For plots in Additional file 1: Figure S11, parameters used were -I 2000 -O 1000 -w 20.

### sRNA analysis

Mapped 24-nucleotide sRNA reads from Col and Cvi embryos collected 6 days after pollination (Robert Erdmann and Mary Gehring, unpublished data) and from Col young and mature leaves (GEO accession number GSE55151) [38] were obtained. The genomecov function in the bedtools suite was used to obtain per-position coverage information. Counts were normalized by converting to RPM by dividing the coverage at each position by [Total reads in the library]/1,000,000. The per-position data were intersected with genic CG positions, and the average of the RPM at both positions was used as the final value for that CG.

### Comparison of [CG] and GC content at dynamic cytosines versus background

To determine whether [CG] or percentage GC content significantly differed around RIL gain or loss sites compared to all potential RIL gain or loss sites, 10,000 random subsets of n = (Number of RIL gain or RIL loss sites) were drawn from the RIL gain or RIL loss background. For each subset, average [CG] or percentage GC content was calculated and then compared to the average value from true RIL gain and RIL loss sites. If fewer than 100 out of the 10,000 random subsets had average [CG] or percentage GC content greater than (right tail) or less than (left tail) the true value, then the true RIL gain or RIL loss sites were considered significantly different from background with *p* < 0.001.

### Motif analysis

DREME [39] was used to identify motifs significantly enriched around RIL gain and RIL loss sites compared to potential RIL gain or potential RIL loss sites (defined as ≤60% and ≥40% methylation in the parent line, respectively; see “Estimating rate of gain and loss of genic methylation in the RILs”). We obtained all RIL gain or loss sites and an equal number of randomly selected potential RIL gain or loss sites to use as a control. We obtained sequences corresponding to 100 bp upstream and downstream of each CG from TAIR10, then ran DREME using the sequences from RIL gain or loss sites as the positive sequence file (-p), the sequences from the subset of potential RIL gain or loss sites as the negative sequence file (-n), with the options -dna -e 0.01. Similarly, to identify motifs enriched around methylated CGs in the parent lines (defined as ≥40% methylation), these CGs were compared to all CGs with data in the parent lines. Because DREME is extremely slow for large numbers of input sequences, any analysis where the positive sequence file contained more than 50,000 sequences was instead performed by drawing three different random subsets of size n = 50,000 from the positive sequence file and running DREME separately on those three subsets matched to equal size subsets drawn randomly from the negative sequence file.

### Locus-specific bisulfite-PCR

DNA was bisulfite treated using the Epigentek BisulFlash bisulfite conversion kit (catalog number P-1054) and PCR amplified (primers listed in Additional file 1: Table S4). Products were purified using the Bioneer AccuPrep PCR purification kit (catalog number K-3034) and cloned into TOPO or pJET and bacteria were grown O/N on selective plates. PCR products from colony PCR were purified using exo-SAP and sequenced. Sequences were aligned to a reference sequence using SeqMan Pro, and Cymate [49] was used to produce methylation plots in Additional file 1: Figure S6.

### RNA-seq

Leaf tissue (100 mg) was harvested from three individual 3-week-old rosettes for RILs 124 and 242, as well as both parent lines. All plants were grown together under the same conditions and harvested at the same time. Tissue was pulverized using the Qiagen TissueLyser II, and RNA was extracted using the RNeasy plant mini kit (Qiagen, catalog number 74903). Libraries were constructed from 1 μg RNA using the RNA Truseq stranded library kit (Illumina) with 15 cycles of amplification. Reads were sequenced on an Illumina HiSeq2000 using a 40-bp single-end protocol. Reads were quality filtered using trim_galore [47] with parameters --phred64 -a ACACTCTTTCCCTACACGACGCTGTTCCATCT --stringency 3 -q 25 and all other parameters default. Filtered reads were mapped to the Col–Cvi metagenome (see “Bisulfite sequencing” section above) using TopHat v2.0.13 [50] with parameters --phred64-quals --library-type fr-firststrand --segment-length 20 -i 70 -I 10000 --read-edit-dist 2 -N 1 with a Bowtie2 (v.2.2.5.0) installation. Additionally, a GTF file of the Araport11 annotations of Col-0 [51] was used with -G to improve junction mapping. To reformat this file for use with the metagenome, the original GTF file was appended to itself, and chromosomes were renamed to match the metagenome. Ambiguously mapped reads (defined as mapQ <5) were remapped to TAIR10 using the same TopHat parameters as previously. FPKM values for genes in each sample were obtained using Cufflinks [52] with parameters --library-type fr-firststrand -m 20 and all others default. We also provided a GTF file of the Araport11 annotations [51] to Cufflinks with the -G option.

### PCA analysis of RNA-seq data

We obtained read counts in each gene for each sample using htseq-count v.0.6.1p1 [53], with parameters -s reverse -a 10 -t exon -i gene_id -m union. We then loaded the count data for all samples into DESeq2 [54] using DESeqDataSetFromMatrix and applied the rlog transformation. PCA was performed on the resulting data using plotPCA over all genes.

### Logistic regression model fitting

We defined 26 models consisting of different combinations of 13 predictors that could potentially influence the probability that a particular CG site will switch its methylation status between the parental generation and F_9_ RIL generation (Fig. 5a). These models were tested separately on each RIL, and separately for CGs in Col- and Cvi-derived regions. To test the ability to predict gain of methylation in the RIL, all genic CGs with ≤60% methylation in the parent line were obtained. We then dropped all CGs with missing data in one or more of the predictors in the model to be tested. Note that observed values of zero (e.g., 0 RPM of sRNAs at a locus) were not considered missing values, and only methylation-related predictors (e.g., local CG methylation, methylation of other parent, variability among strains) had the potential to have missing values. Because of the physical proximity of some CGs to others, the raw data have a high degree of autocollinearity. To reduce this, we sampled the data such that no two CGs in the data were within 200 bp of each other. We then counted the number of successes (CGs where the RIL gained methylation relative to the parent line) remaining in the data, and randomly sampled the same number of failures, to obtain a subset of the data where 50% of the observations are successes and 50% are failures, and all observations are ≥200 bp apart. We fit a logit model to these data (see logit command from Stata [55]), then evaluated the model by using it to predict success and failure for a second subset, obtained as previously described, and calculating the percentage of CGs correctly classified (see Estat classification command from Stata [55]). Because half the observations in the dataset are successes by design, a naïve predictor (that randomly guesses success or failure) will be correct 50% of the time. We repeated this analysis ten times for each RIL, obtaining 100 total estimates of prediction accuracy for each model. These 100 observations were used to construct each boxplot shown in Fig. 5. Similarly, to predict loss of methylation in the RILs, we obtained all CGs with ≥40% methylation in the parent line, then proceeded as described for gain of methylation. Stata code used for this analysis is provided in predict_logit_train_test.do, and the full dataset used in this analysis is provided in full_dataset.txt (see “Availability of data and materials”). Code to perform a simplified version of this analysis using only local methylation to predict methylation variability across strains (Additional file 1: Figure S11) is provided in predict_logit_train_test_mini.do (see “Availability of data and materials”).

### Analysis of *Z. mays* and *B. distachyon* methylation data

We downloaded bisulfite-sequencing reads for five maize accessions (B73, Mo17, CML322, Oh43, and Tx303) published in [43] from the SRA (accession numbers SRR850328, SRR850332, SRR1610959, SRR1610960, and SRR1610961, respectively). *B. distachyon* reads for seven inbred lines [42] were also obtained from the SRA (Bd21, SRR1972494; Bd21-3, SRR1972495; Bd1-1, SRR1972498; Bd3-1, SRR1972496; Bd30-1, SRR1972497; BdTR12C, SRR1972499; Koz3, SRR1972500). Maize reads were mapped to the B73 reference genome version 2, and *B. distachyon* reads were mapped to the Bd21 v2 reference genome. All datasets were mapped using the same pipeline and parameters as for the *A. thaliana* RIL bisulfite-sequencing data (see above). Data for CGs on both strands were combined as above, and all CGs with data missing in no more than one strain were classified into three categories using the same approach noted above, except because of the small number of strains, the three “bimodal” categories were combined with the “distributed” class (collectively referred to as “variable” in the text). Regression analysis proceeded as above, except “successes” were defined as all CGs classified as “variable,” and the background (“failures”) were defined separately as either all “unimodal lo” CGs or all “unimodal hi” CGs (Additional file 1: Figure S11). Code to perform the regression analysis for maize is provided in predict_logit_train_test_maize.do (see “Availability of data and materials” section), and the full dataset used in this analysis for B73 is provided in full_maize_B73_data.txt (see “Availability of data and materials”). Code to perform the regression analysis for *B. distachyon* is provided in predict_logit_train_test_distachyon.do, and the full dataset used in this analysis for Bd1-1 is provided in full_distachyon_Bd1-1_data.txt (see “Availability of data and materials”).

## Additional file

Additional file 1: Supplemental figures and tables. **Figures S1**–**S12** and **Tables S1–S4**. (PDF 12503 kb)

## Abbreviations

Col: *A. thaliana* reference strain Columbia or Col-0
Cvi: *A. thaliana* strain Cape Verde Islands
PCA: Principal component analysis
RdDM: RNA-directed DNA methylation
RIL: Recombinant inbred line
RIL gain sites: CG sites that are more methylated in the RIL than in the parental line for the CG site
RIL loss sites: CG sites that are less methylated in the RIL than in the parental line for the CG site
sRNA: Small RNA
TE: Transposable element
