# Supplementary material for: Proximal methylation features associated with nonrandom changes in gene body methylation
Source: Genome Biol. 2017 Apr 26;18:73. doi: 10.1186/s13059-017-1206-2 (PMC5406939; doi:10.1186/s13059-017-1206-2)
Supplement: Additional file 1: — Supplemental figures and tables. Figures S1–S12 and Tables S1–S4. (PDF 12503 kb) [file 13059_2017_1206_MOESM1_ESM.pdf]

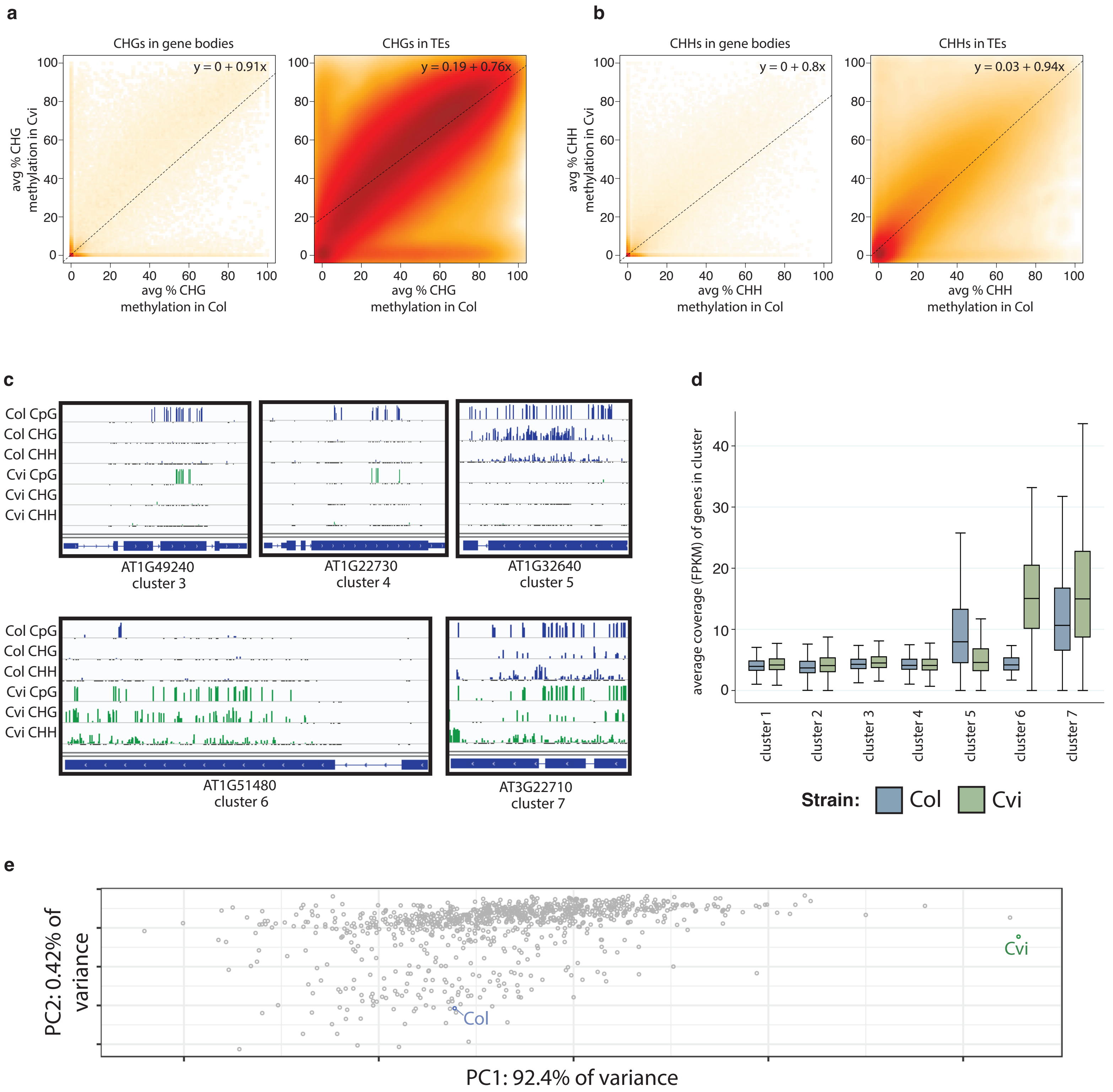

Figure S1. Further characterization of gene body methylation in Cvi. a,b) Smoothed scatterplots of average methylation at individual CHGs (a) and CHHs (b) in Col *vs.* Cvi, in genes (left) and TEs (right). Line of best fit obtained using the R lm() function. **c**) Examples of genes from 5 different clusters from Fig. 1b. Methylation data for the first Col and Cvi replicate shown on a scale from 0%-100% methylation. Ticks below the x-axis indicate methylation levels of zero, while the absence of any mark indicates a lack of data at that position. **d**) Boxplot of average depth across the two Col BS-seq replicates (blue) and the two Cvi BS-seq replicates (green) for genes in the seven clusters from Fig. 1b. **e**) PCA of weighted average gene body methylation for 927 strains from the 1001 methylomes project [19].

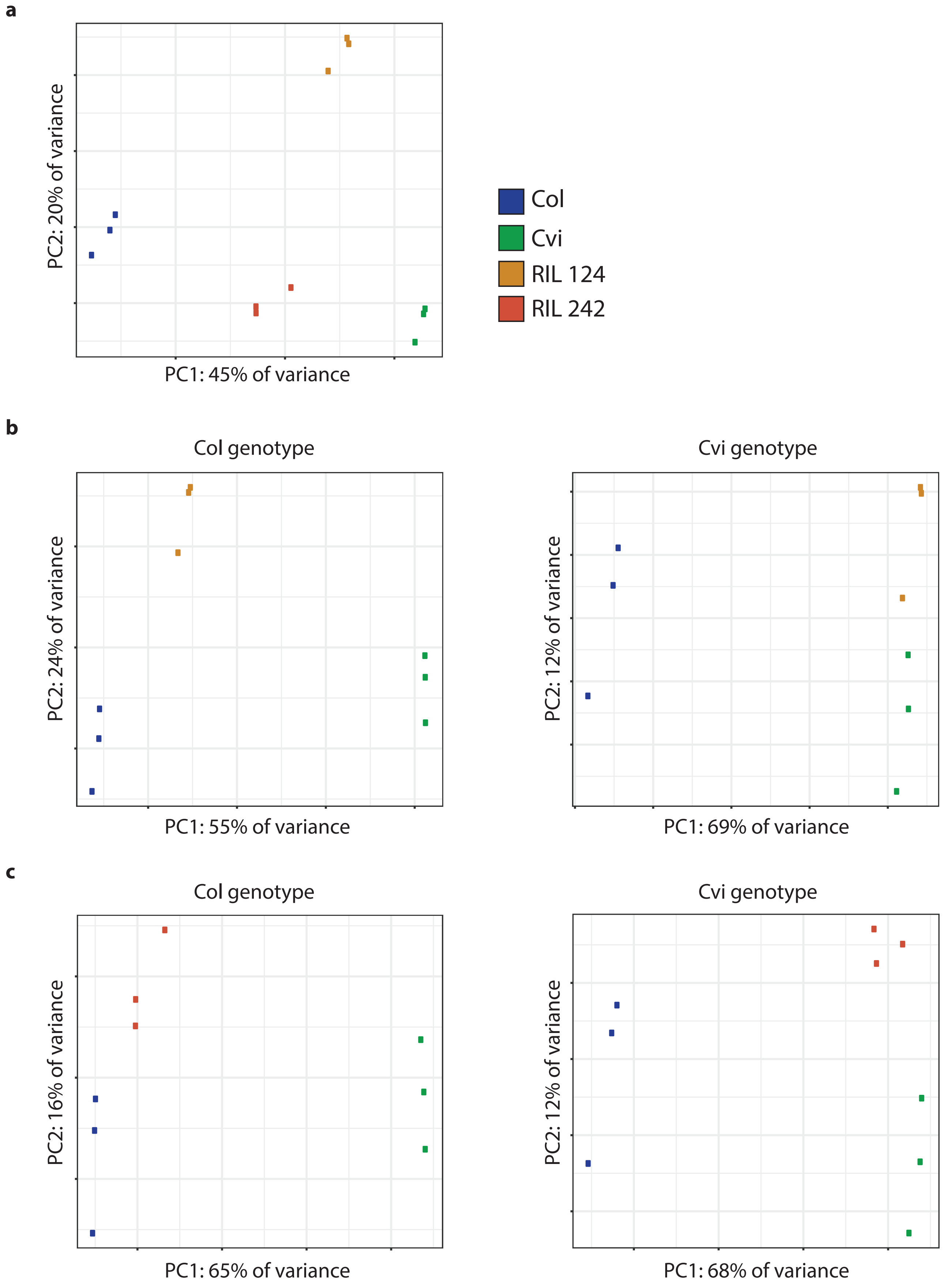

Figure S2. PCA of gene expression levels in two RILs and the Col and Cvi parent lines. a) PCA over all genes for all samples (3 replicates each of Col, Cvi, RIL 124 and RIL 242). **b)** PCA over genes in RIL 124, performed separately for genes with Col genotype (left) and Cvi genotype (right). **c)** Same as **b**, but for RIL 242.

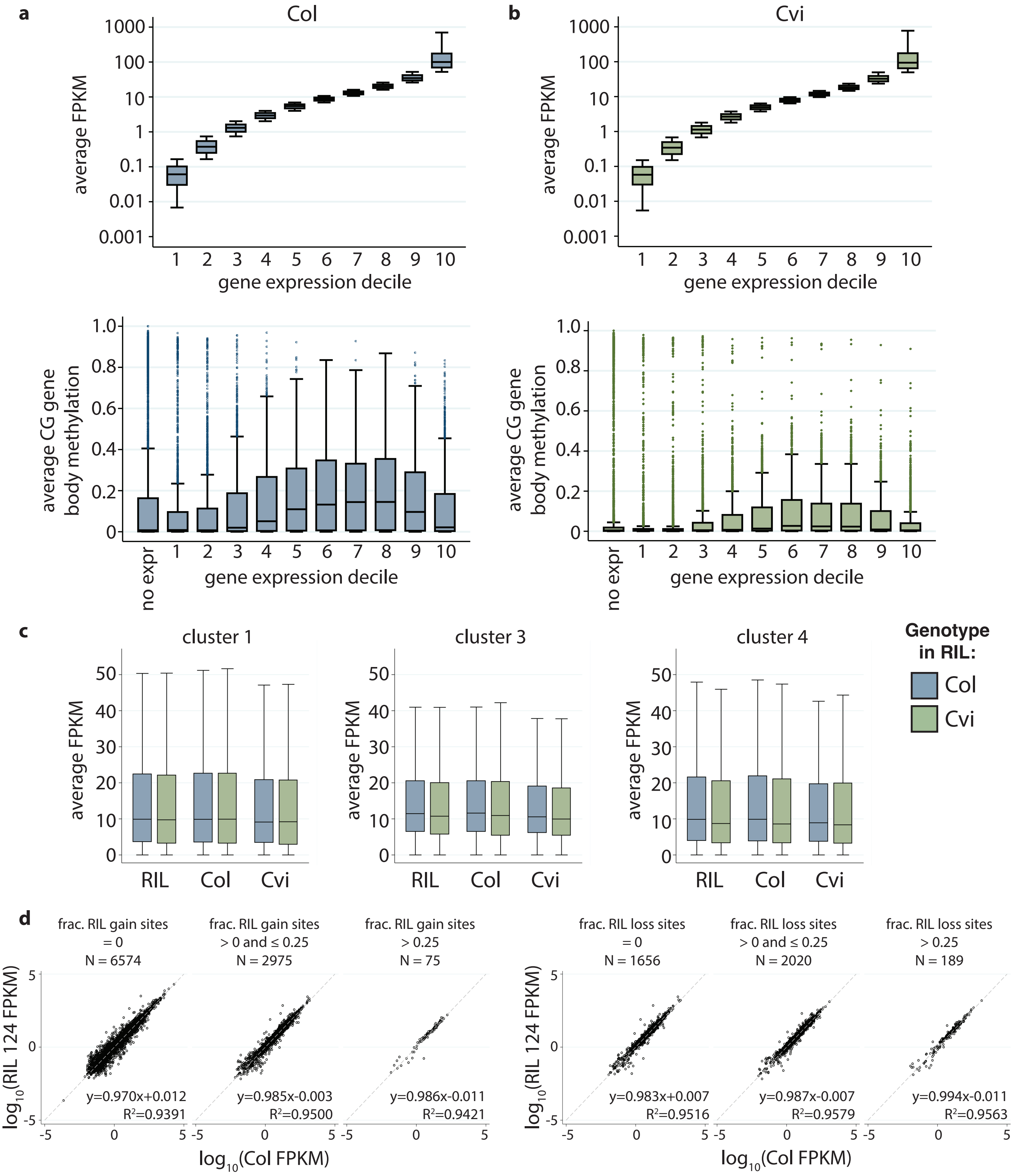

Figure S3. Relationship between gene body methylation and gene expression. a,b) Average FPKM (top) and weighted gene body CpG methylation (bottom) for genes in different expression deciles in Col (**a**) and Cvi (**b**). Genes with no read support were separated into their own category. **c**) Distribution of average FPKM values across all replicates in Col, Cvi and RIL 242, for genes in clusters 1, 3 and 4 from Fig. 1b. Genes in each cluster have been separated according to their genotype in RIL 242. **d**) For genes inherited from Col in RIL 124, comparison of average FPKM in Col *vs.* RIL 124, by fraction RIL gain (left) or RIL loss (right) sites. RIL gain sites = CGs unmethylated in the parent line that become methylated in the RIL; RIL loss sites = CGs methylated in the parent line that become unmethylated in the RIL. Fraction RIL gain or RIL loss is calculated using the total number of CGs with the potential to become RIL gain or RIL loss sites based on methylation level in Col parent. Analysis limited to genes with at least 5 potential RIL gain (left) or 5 potential RIL loss (right) sites. Similar results were obtained regardless of parental genotype and in RIL 242. A small number of genes with log(FPKM) outside the interval [-5,5] or with no detectable expression (FPKM = 0) were omitted.

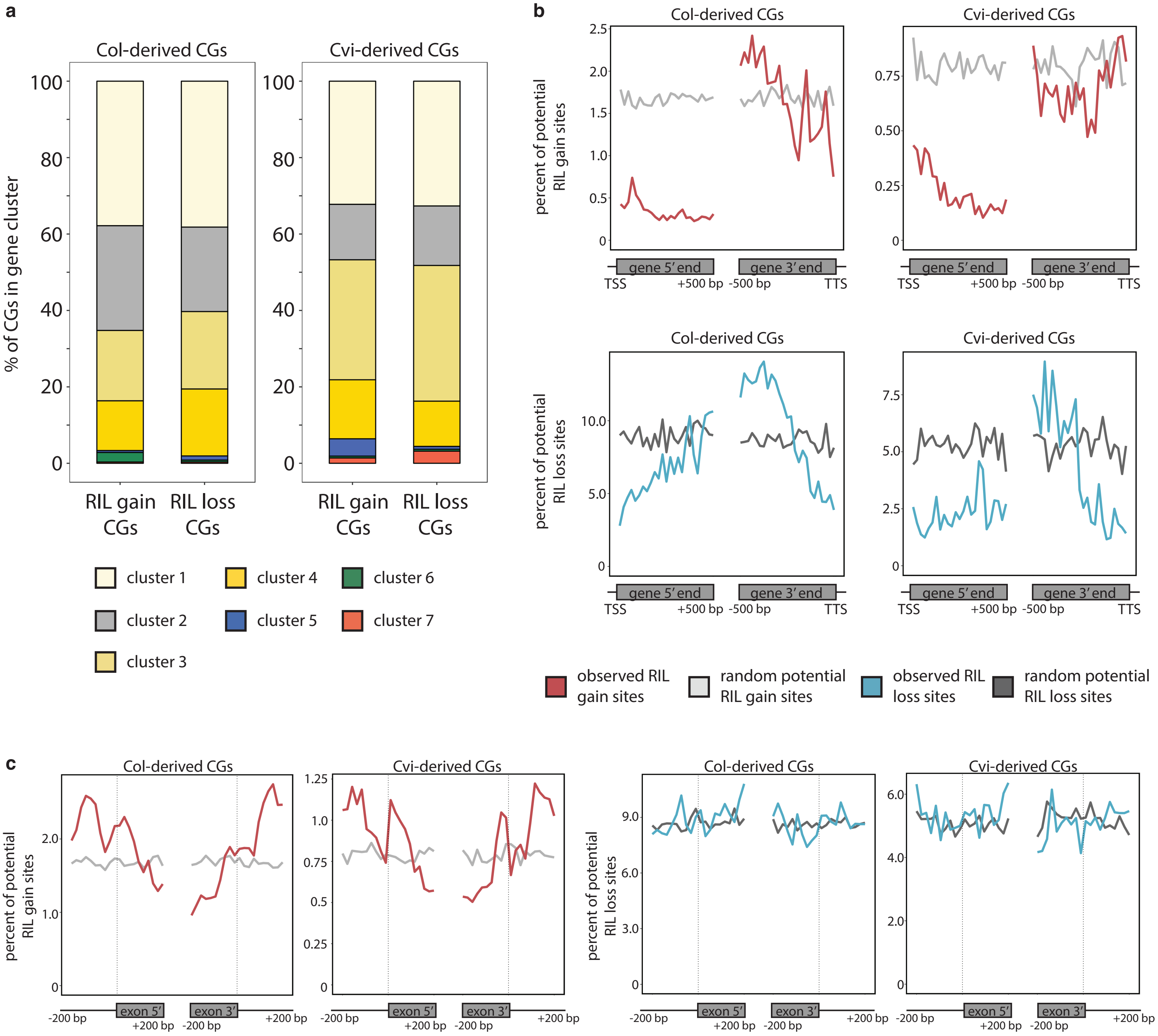

Figure S4. Distribution of dynamic cytosines in genes, exons and introns. a) Distribution of RIL gain and RIL loss sites among the 7 clusters from Fig. 1b, for CGs in RIL 8 inherited from Col (left) and CGs inherited from Cvi (right). RIL gain sites = CGs unmethylated in the parent line that become methylated in the RIL; RIL loss sites = CGs methylated in the parent line that become unmethylated in the RIL. **b)** Distribution of RIL gain and RIL loss sites around gene TSS and TTS, for CGs inherited from Col (left) or Cvi (right). For comparison, an equal number of CGs was drawn from the set of all potential RIL gain sites (light gray) or all potential RIL loss sites (dark gray) and the distribution of these random CGs was plotted alongside the true distribution of RIL gain or RIL loss sites. Plots were first created for each of the 10 RILs separately, then percents at each x-coordinate for each of the 10 RILs were averaged to produce a “consensus” plot representing average behavior across all RILs (shown here). **c)** Same as **b**, but for CGs within and flanking exons.

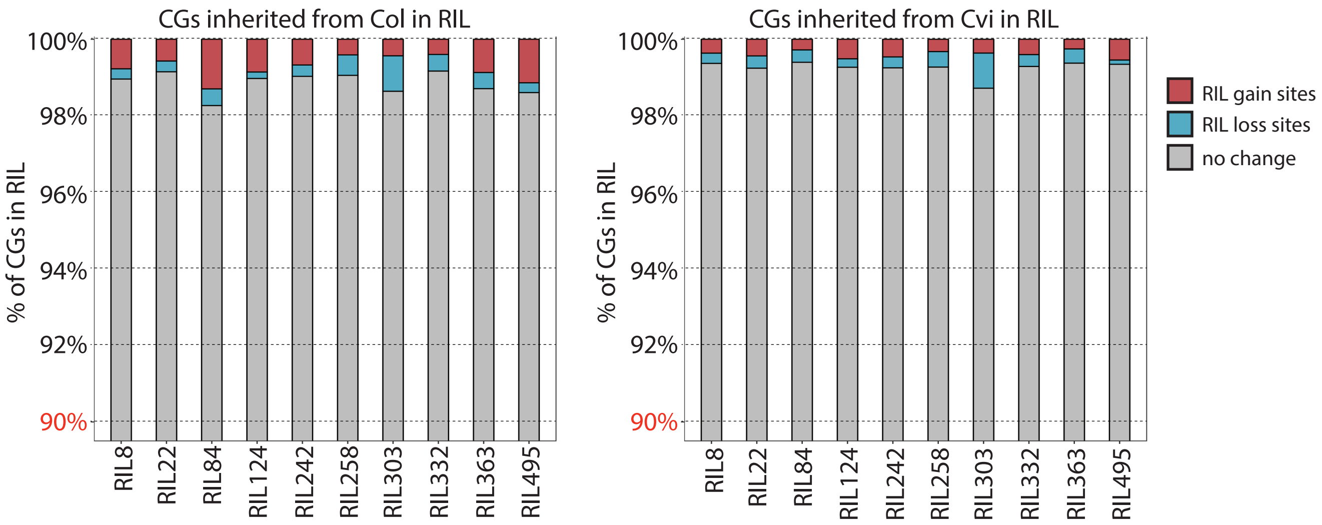

Figure S5. CGs that fail to maintain the parental methylation state in TEs. Summary of the fraction of CG sites in TEs that failed to maintain the parental methylation state in each RIL. Note that y-axis begins at 90%.

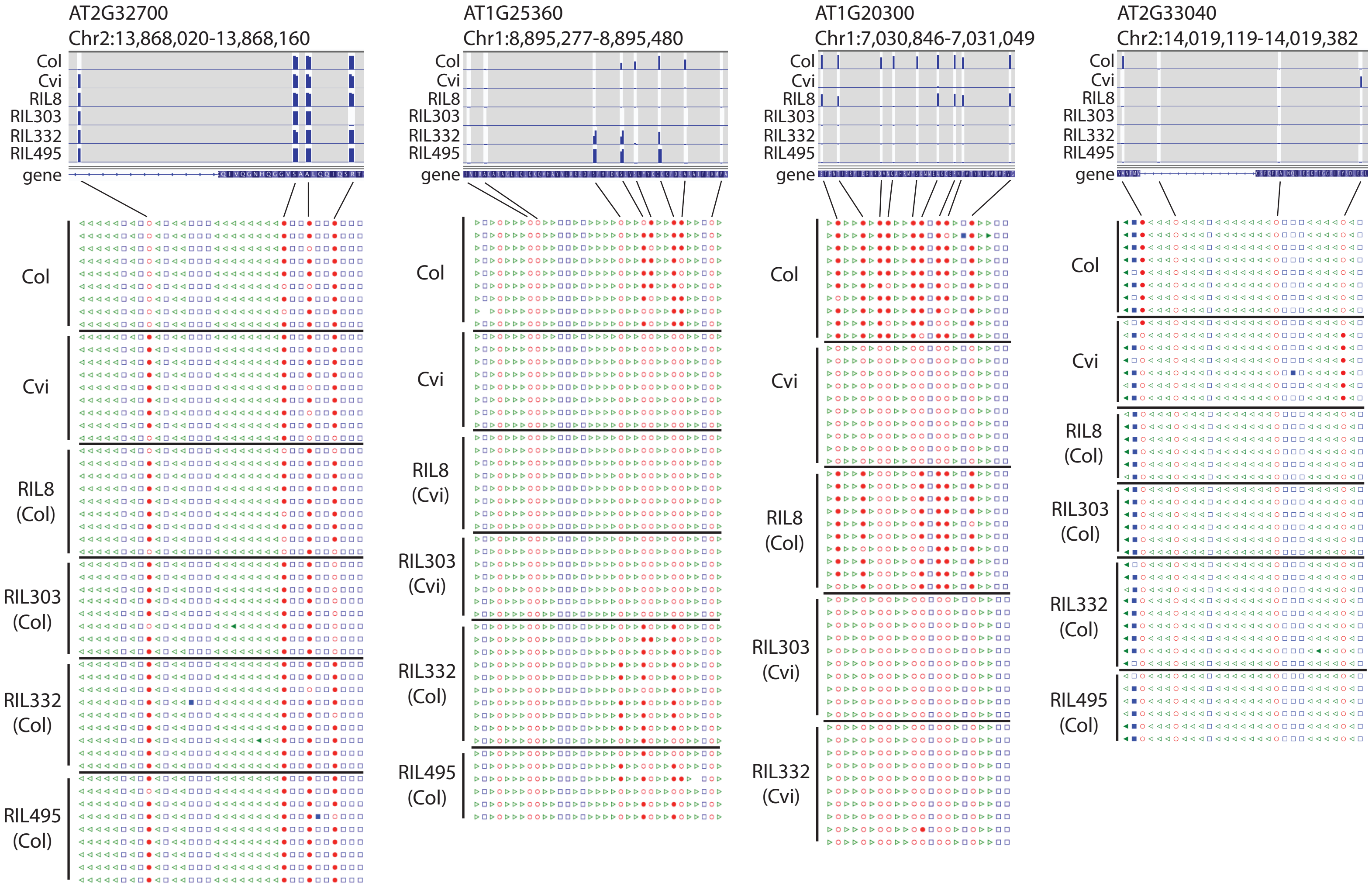

Figure S6. BS-PCR validation of dynamic and stable cytosines. Validation of four loci containing at least one dynamic cytosine. Methylation profiles from whole-genome bisulfite-sequencing data shown at the top of each locus along with a gene track, with lines indicating the corresponding CG in BS-PCR results. Genotype for RILs at each locus shown below RIL label.

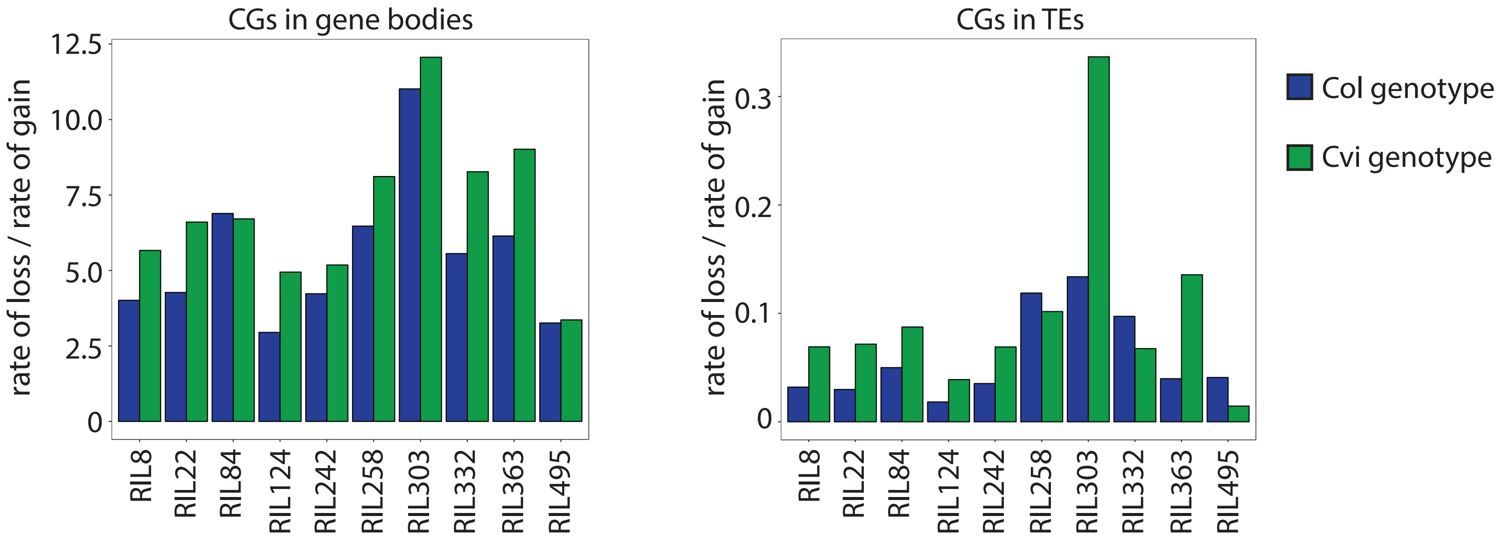

Figure S7. Ratio of the rate of loss to the rate of gain of methylation in each RIL. For each line, the ratio of the rate of methylation loss to the rate of methylation gain is shown for sites inherited from Col (blue) or Cvi (green), in gene bodies (left) or TEs (right). Note that y-axes of each plot use different scales.

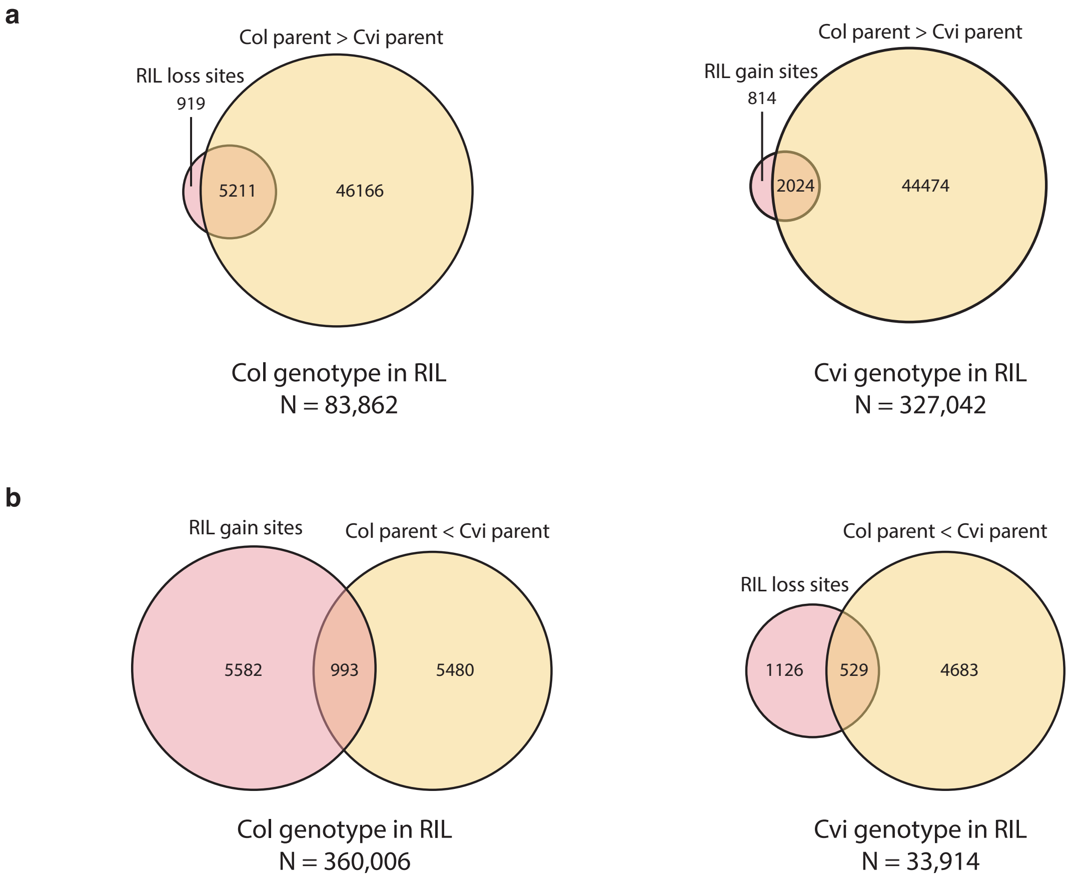

Figure S8. Dynamic cytosines occur at CGs that are differentially methylated between Col and Cvi. a) For CG sites in gene bodies with Col genotype in RIL 8, the overlap between sites that lost methylation in the RIL and sites where Cvi is less methylated than Col (left). For CGs in gene bodies with the Cvi genotype in RIL 8, overlap between sites that gained methylation in the RIL and sites where Col is more methylated than Cvi (right). Number indicated below genotype is the total number of genic CGs from the indicated parent line in the RIL with the potential to become RIL gain/loss sites based on methylation levels in the parent lines. For RIL gain sites, this was CGs with ≤ 60% methylation in the parent line, and for RIL loss this was ≥ 40% methylation. This number indicates total population size used for the hypergeometric test. **b)** Similar to **a**, but for sites where Cvi is more methylated than Col.

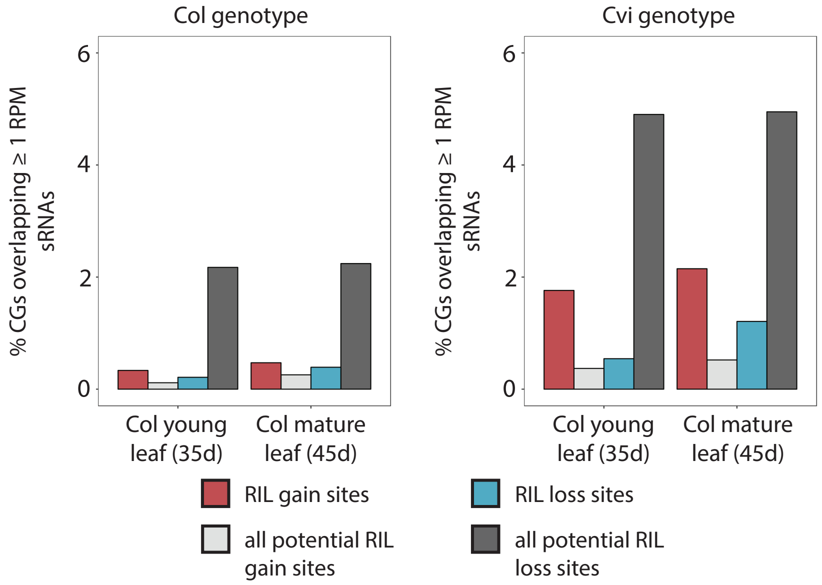

Figure S9. Leaf sRNA abundance around dynamic cytosines. Percent of indicated genic CGs from Col- (left) and Cvi- (right) derived regions in the RILs overlapping at least 1 RPM of 24 nt sRNAs from young (35 days old) or mature (45 days old) Col leaves [38].

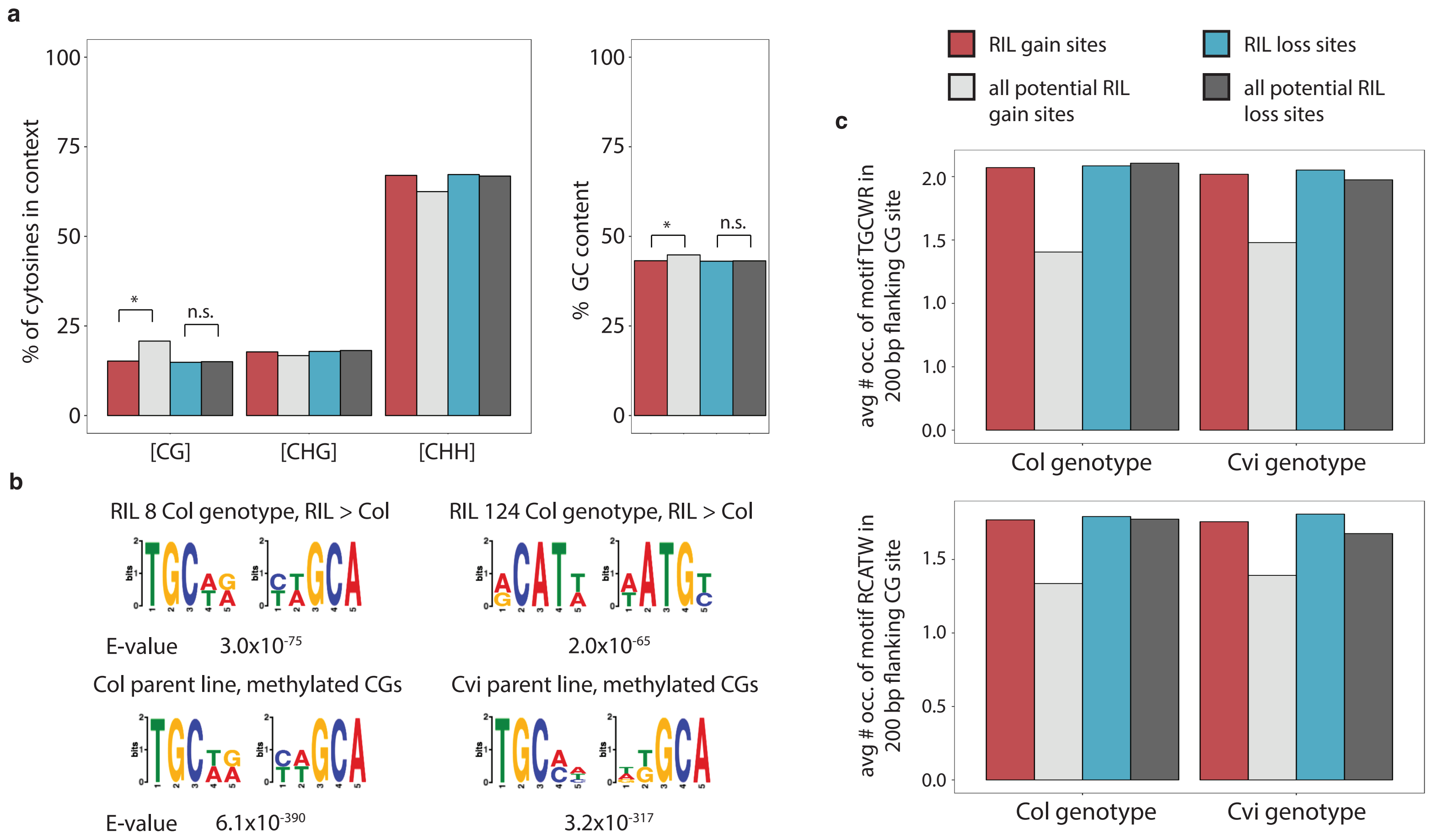

Figure S10. Sequence features around RIL gain and loss sites. a) % of cytosines in the CG, CHG and CHH contexts ([CG], [CHG] and [CHH]) and overall %GC content in the 200 bp surrounding indicated CGs. Data shown for CGs in RIL 8 inherited from Col. * = $p < 0.001$ (see methods). **b)** Top sequence motifs enriched in the 200 bp surrounding CG sites that have gained methylation in RILs (motifs for 2 RILs shown). Top sequence motifs enriched in the 200 bp surrounding methylated CGs (≥ 40% methylation) in Col or Cvi. **c)** Average # of times motif TGCWR (top) and RCATW (bottom) occur in 200 bp surrounding indicated CGs (legend same as **a**).

a

Model #	1	2	3	4	5	6	7
local CG methyl.	x			x	x		x
local CHG methyl.		x		x		x	x
local CHH methyl.			x		x	x	x

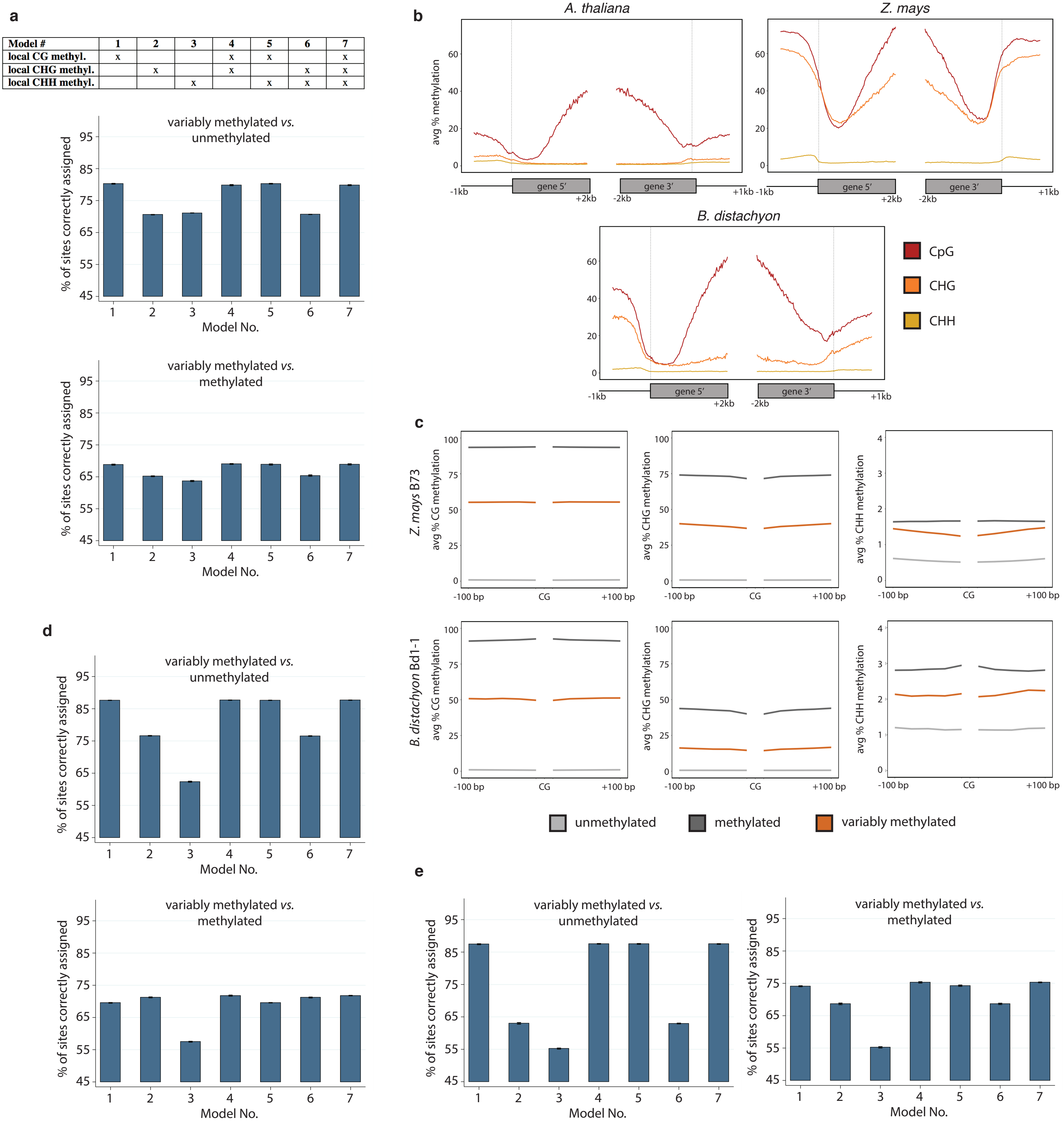

Figure S11. Variably methylated CGs in maize and *B. distachyon* are associated with intermediate methylation levels. a) % of CGs correctly assigned when using indicated models to predict variably methylated CGs against a background of invariably unmethylated CGs (top) or against a background of invariably methylated CGs (bottom), using local methylation data from Col/Cvi RIL 8. Combination of predictors used for each model indicated in table at top of panel. Error bars indicate +/- 1 s.d. from 10 randomly drawn background datasets (see methods). **b)** Distribution of CG, CHG and CHH methylation in gene bodies in *A. thaliana* Col-0 (left) and *Zea mays* B73 (right) [43] and *B. distachyon* Bd1-1 (bottom) [42]. **c)** Distribution of CG (top), CHG (middle) and CHH (bottom) methylation around genic CGs in *Z. mays* B73 (top) and *B. distachyon* Bd1-1(bottom). CGs were first classified into 3 categories based on methylation variability across 5 maize strains [43] or 7 *B. distachyon* strains [42]. **d)** Analysis in part **a** repeated using data from *Z. mays* (see methods). Models same as **a**. **e)** Analysis in part **a** and **d** repeated using data from *B. distachyon* (see methods). Models same as **a**.

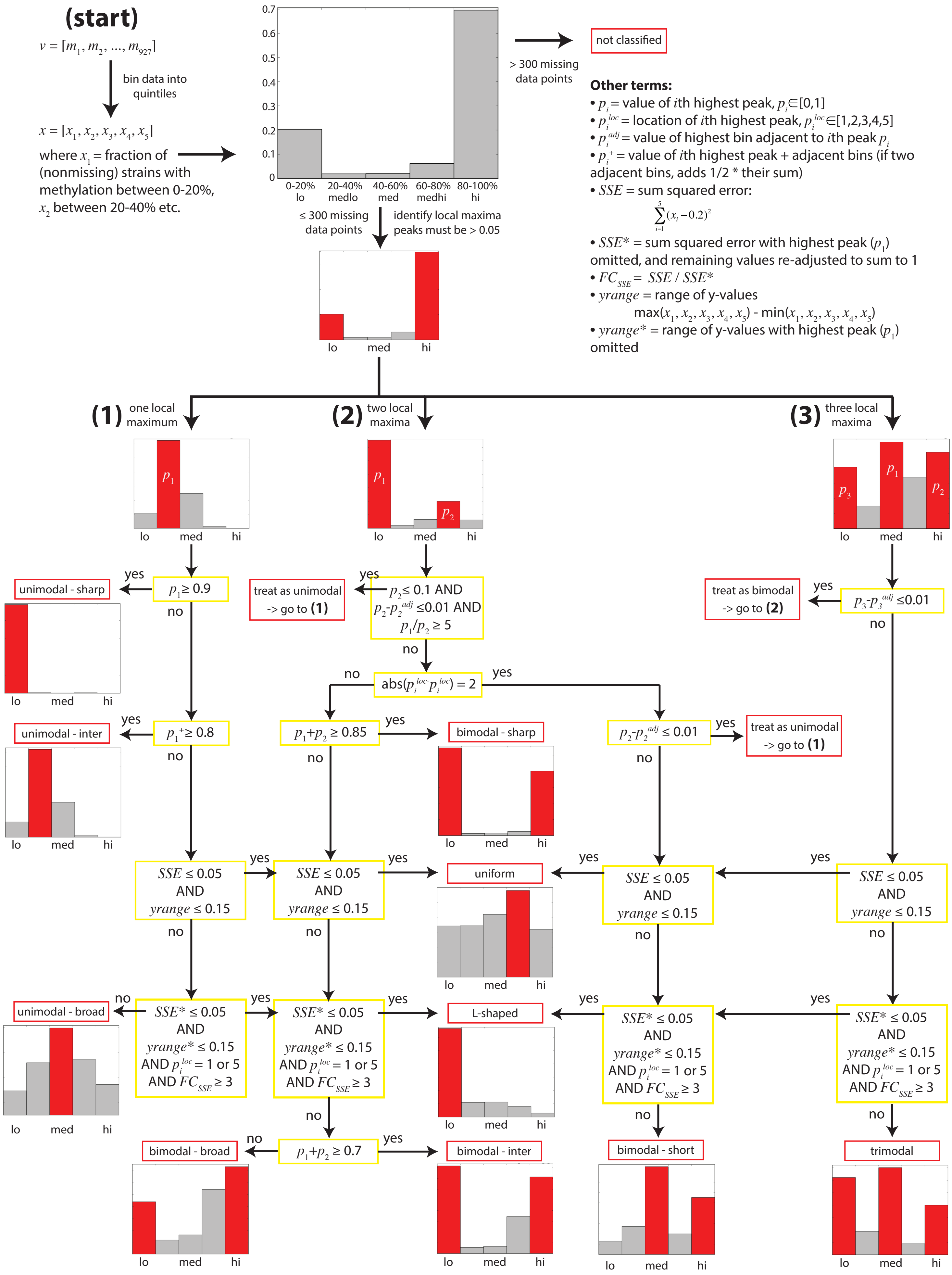

Figure S12: Flowchart describing method used to classify CGs based on the variability of methylation levels across 927 wild-type Arabidopsis strains. Let $v = [m_1, m_2, \ldots, m_{927}]$ be a vector of length 927 containing the % methylation at a specific CG for all 927 different strains from [19]. Begin flowchart at (start) position above.

Table S1: Bisulfite-seq samples used in this study and overall methylation data

Overall methylation data

sample name	CpG Context				CHG Context				CHH Context				Chloroplast methylation		
	# sites with >= 1 overlapping read	# sites with >= 5 overlapping reads	average %me over sites with >= 5 reads coverage	average depth for sites with >= 1 overlapping read	# sites with >= 1 overlapping read	# sites with >= 5 overlapping reads	average %me over sites with >= 5 reads coverage	average depth for sites with >= 1 overlapping read	# sites with >= 1 overlapping read	# sites with >= 5 overlapping reads	average %me over sites with >= 5 reads coverage	average depth for sites with >= 1 overlapping read	avg. CpG methylation	avg. CHG methylation	avg. CHH methylation
Col_1	3669845	1795633	39.92%	9.36	4067134	1951287	14.85%	8.88	17993438	7466925	5.06%	7.53	0.45%	0.50%	0.52%
Col_2	4373940	2469353	36.70%	9.81	4854024	2743703	12.40%	9.49	22498806	11088708	4.08%	8.14	0.42%	0.42%	0.44%
Cvi_1	3350437	1610412	29.42%	9.17	3742294	1754054	14.49%	8.62	16358992	6668461	5.20%	7.53	1.67%	1.12%	0.59%
Cvi_2	3790285	2002885	27.22%	9.42	4243265	2222805	12.93%	9.00	19117190	8747558	4.46%	7.91	1.12%	0.82%	0.52%
8_1	4106053	2320118	31.59%	10.46	4584376	2595389	12.35%	10.14	21021396	10420517	4.59%	8.81	0.78%	0.64%	0.49%
8_2	4042725	2258348	31.78%	10.32	4508645	2506626	12.69%	9.95	20605833	10032739	4.88%	8.64	1.00%	0.77%	0.51%
22_1	3212133	1320298	36.15%	6.92	3531182	1396715	16.07%	6.57	15204676	5139640	5.45%	5.78	1.01%	0.80%	0.49%
22_2	3354978	1399906	36.38%	7.11	3696690	1490625	16.18%	6.77	16107583	5576622	5.37%	6.01	0.87%	0.72%	0.48%
84_1	3256480	1379998	32.79%	7.38	3588864	1466124	15.57%	7.02	15562625	5454219	6.03%	6.20	1.49%	1.10%	0.67%
84_2	3323935	1404303	32.93%	7.27	3664964	1494378	15.51%	6.93	15985212	5593433	6.21%	6.15	1.29%	0.96%	0.58%
124_1	4234811	2588272	32.03%	15.21	4689833	2833137	12.95%	14.60	21644469	11429772	5.29%	12.21	0.48%	0.44%	0.44%
124_2	3874914	2067098	34.61%	11.42	4287645	2238684	14.63%	10.91	19333128	8753255	6.26%	9.32	0.51%	0.45%	0.43%
242_1	4150337	2448973	30.82%	13.67	4598541	2673742	10.44%	13.08	21102063	10672691	5.10%	10.97	1.04%	0.78%	0.57%
242_2	3994277	2249110	32.04%	12.85	4420714	2442651	11.02%	12.23	20128058	9682121	5.24%	10.33	0.95%	0.72%	0.56%
258_1	3362989	1478653	33.32%	7.59	3707867	1577528	14.38%	7.28	16156033	5904685	6.51%	6.40	0.46%	0.44%	0.45%
258_2	3336451	1404803	34.39%	7.10	3679368	1499140	14.26%	6.82	16038033	5597419	5.82%	6.02	0.41%	0.43%	0.42%
303_1	3815699	2007050	32.96%	9.87	4249516	2206906	14.32%	9.47	19171932	8727925	5.58%	8.24	1.25%	0.98%	0.75%
303_2	4058136	2310802	31.44%	10.94	4524634	2565467	13.13%	10.60	20757134	10348829	4.97%	9.18	0.82%	0.64%	0.45%
332_1	3734524	1712791	35.15%	7.50	4161031	1884012	13.67%	7.15	18661095	7301114	5.04%	6.32	0.42%	0.36%	0.35%
332_2	3821707	1809981	34.86%	7.76	4262076	2004343	13.56%	7.43	19236606	7840157	4.94%	6.56	0.46%	0.39%	0.39%
363_1	3246670	1384556	32.27%	7.60	3580819	1466889	13.90%	7.18	15490889	5427072	5.49%	6.29	1.18%	0.92%	0.70%
363_2	3339804	1398410	32.46%	7.12	3687469	1488425	13.76%	6.78	16125081	5567354	5.53%	6.02	1.02%	0.75%	0.52%
495_1	3580659	1657984	36.17%	7.74	4003395	1834837	14.51%	7.46	17866595	7151511	6.59%	6.63	0.55%	0.46%	0.46%
495_2	3560268	1521432	37.46%	6.80	3981982	1684145	14.68%	6.56	17799153	6529027	6.22%	5.88	0.42%	0.37%	0.36%

Alignment Data

sample name	barcode	date(s) sequenced	read length	total pairs sequenced	pairs passed QC	pairs uniquely mapped to metagenome	pairs uniquely mapped to reference genome	total pairs uniquely mapped	total pairs remaining after removing PCR dup	single reads uniquely mapped (PCR dedup)
Col_1	ATCACG	3/11/16, 03/31/16,04/13/16	40x40, 100x100, 50x50	38715299	36661544	4070182	13289345	17359527	9124227	2871588
Col_2	TTAGGC	3/11/16, 03/31/16,04/13/16	40x40, 100x100, 50x50	38256538	36095183	4561851	14609983	19171834	11086036	3616170
Cvi_1	CGATGT	3/11/16, 03/31/16,04/13/16	40x40, 100x100, 50x50	39904327	37785311	4451199	12344974	16796173	7962301	155615
Cvi_2	GATCAG	3/11/16, 03/31/16,04/13/16	40x40, 100x100, 50x50	38692279	36457421	4264395	12561152	16825547	9142500	154716
8_1	TGACCA	3/11/16, 03/31/16,04/13/16	40x40, 100x100, 50x50	44842727	42228122	4904867	15935305	20840172	11205989	2052691
8_2	TAGCTT	3/11/16, 03/31/16,04/13/16	40x40, 100x100, 50x50	40875794	38589860	4846897	14899799	19746696	10860115	2012262
22_1	ATCACG	12/9/16	40x40	30592479	29201259	3186739	12981996	16168735	9332372	1315867
22_2	ACAGTG	12/9/16	40x40	34970108	33427227	3561611	14535436	18097047	10005530	1408436
84_1	CGATGT	12/9/16	40x40	35887742	34274726	3828984	14506769	18335753	10007175	1152309
84_2	GCCAAT	12/9/16	40x40	35937164	34310689	3789821	14459898	18249719	10112898	1165392
124_1	GCCAAT	10/3/14	40x40	113178957	105999473	11214935	46878329	58093264	27890189	3628222
124_2	CTTGTA	10/3/14	40x40	73284286	68660780	7646079	29752584	37398663	18853649	2511528
242_1	TAGCTT	10/3/14	40x40	98784225	92366676	9471647	39638312	49109959	24480158	3207840
242_2	GGCTAC	10/3/14	40x40	111197919	104054525	10088621	42118216	52206837	21782756	2920509
258_1	TTAGGC	12/9/16	40x40	34187535	32735322	4030112	15337402	19367514	10800436	1372871
258_2	CAGATC	12/9/16	40x40	32198281	30806273	3494639	14172380	17667019	10056285	1253957
303_1	CAGATC	3/11/16, 03/31/16,04/13/16	40x40, 100x100, 50x50	37558572	35453971	4577405	13741875	18319280	9725249	1707899
303_2	GGCTAC	3/11/16, 03/31/16,04/13/16	40x40, 100x100, 50x50	43862802	41380871	5189733	16200162	21389895	11465611	1999511
332_1	ACAGTG	3/11/16, 03/31/16,04/13/16	40x40, 100x100, 50x50	28768466	27130047	3096478	9790863	12887341	7248655	1379823
332_2	GCCAAT	3/11/16, 03/31/16,04/13/16	40x40, 100x100, 50x50	30722094	29045093	3254566	10142496	13397062	7530647	1447501
363_1	TGACCA	12/9/16	40x40	38326100	36569425	3973864	15450620	19424484	10213174	939165
363_2	ACTTGA	12/9/16	40x40	35725091	34190345	3705217	14497620	18202837	9984139	914212
495_1	ACTTGA	3/11/16, 03/31/16,04/13/16	40x40, 100x100	26966736	25511739	3217350	8712657	11930007	6346815	1053647
495_2	CTTGTA	3/11/16, 03/31/16,04/13/16	40x40, 100x100	23779337	22406495	2743325	7634190	10377515	5780911	929683

Table S2: Pearson correlation coefficients between all samples for CG methylation data

	Col 1	Col 2	Cvi 1	Cvi 2	8 1	8 2	22 1	22 2	84 1	84 2	124 1	124 2	242 1	242 2	258 1	258 2	303 1	303 2	332 1	332 2	363 1	363 2	495 1	495 2
**Col 1**	1																							
**Col 2**	0.9803	1																						
**Cvi 1**	0.7475	0.7428	1																					
**Cvi 2**	0.7394	0.7332	0.986	1																				
**8 1**	0.8544	0.8524	0.843	0.8364	1																			
**8 2**	0.8536	0.852	0.8417	0.8352	0.9834	1																		
**22 1**	0.8637	0.8622	0.8504	0.8455	0.8569	0.8559	1																	
**22 2**	0.8607	0.8592	0.853	0.848	0.8591	0.8581	0.98	1																
**84 1**	0.8511	0.8497	0.8547	0.8494	0.8885	0.888	0.8561	0.853	1															
**84 2**	0.8517	0.8499	0.8534	0.8484	0.8874	0.8867	0.8571	0.8537	0.9669	1														
**124 1**	0.8506	0.8485	0.8502	0.8434	0.8842	0.8831	0.8445	0.8449	0.8666	0.8654	1													
**124 2**	0.8542	0.8525	0.8517	0.8455	0.8866	0.8853	0.8472	0.8474	0.8687	0.8675	0.9892	1												
**242 1**	0.8515	0.8493	0.8463	0.8393	0.8689	0.8686	0.8456	0.8421	0.8738	0.8731	0.8584	0.8607	1											
**242 2**	0.8528	0.8508	0.8469	0.8402	0.8693	0.8689	0.8468	0.8434	0.8738	0.8731	0.8589	0.8611	0.987	1										
**258 1**	0.8546	0.8534	0.8472	0.8422	0.8549	0.8538	0.8747	0.872	0.8686	0.8689	0.8955	0.8965	0.8391	0.8391	1									
**258 2**	0.8559	0.8547	0.8497	0.8443	0.8566	0.8556	0.8765	0.8738	0.8703	0.8701	0.8977	0.8988	0.8407	0.8412	0.9764	1								
**303 1**	0.8282	0.8267	0.8729	0.8675	0.8503	0.8491	0.8696	0.8667	0.8683	0.868	0.8651	0.8674	0.8674	0.8679	0.8661	0.8674	1							
**303 2**	0.8268	0.8248	0.8729	0.8672	0.8493	0.8482	0.8701	0.8675	0.8675	0.8674	0.8652	0.8676	0.8669	0.8675	0.8655	0.867	0.9791	1						
**332 1**	0.8584	0.8575	0.8444	0.838	0.8371	0.8364	0.8988	0.9018	0.8336	0.8338	0.8576	0.8606	0.8298	0.8318	0.8499	0.8517	0.8211	0.82	1					
**332 2**	0.8576	0.8564	0.8442	0.8375	0.8359	0.8349	0.8985	0.9015	0.8323	0.8324	0.8574	0.8605	0.8284	0.8306	0.849	0.8509	0.8197	0.8186	0.9808	1				
**363 1**	0.8211	0.8188	0.8953	0.8912	0.8603	0.8592	0.8986	0.8972	0.8894	0.8897	0.8407	0.8428	0.8786	0.8785	0.8546	0.8563	0.8699	0.8703	0.8821	0.8816	1			
**363 2**	0.8198	0.8174	0.8951	0.8911	0.8594	0.8584	0.8986	0.897	0.8884	0.889	0.8398	0.842	0.8781	0.878	0.8534	0.8549	0.8692	0.8694	0.8817	0.8812	0.9818	1		
**495 1**	0.8556	0.8545	0.8468	0.8399	0.8356	0.8346	0.8409	0.8377	0.8756	0.875	0.8999	0.9017	0.8889	0.8892	0.8858	0.8874	0.8607	0.8604	0.8548	0.8543	0.861	0.8603	1	
**495 2**	0.8565	0.8554	0.848	0.8414	0.8375	0.8364	0.842	0.8389	0.8763	0.8755	0.901	0.9024	0.8898	0.89	0.8861	0.8883	0.8614	0.8616	0.8556	0.8552	0.8619	0.8614	0.9855	1

	Correlation between replicates for sample
**Col**	0.9803
**Cvi**	0.986
**RIL8**	0.9834
**RIL22**	0.98
**RIL84**	0.9669
**RIL124**	0.9892
**RIL242**	0.987
**RIL258**	0.9764
**RIL303**	0.9791
**RIL332**	0.9808
**RIL363**	0.9818
**RIL495**	0.9855
**min**	0.9669
**max**	0.9892

Table S3: mRNA-seq samples used in this study

sample	read_len	barcode	Total reads obtained	Reads passed QC	total reads aligned	% reads aligned
col_1	40bp	TGACCA	13221536	13073662	12145817	92.90%
col_2	40bp	CGATGT	8537150	8438963	7841116	92.90%
col_3	40bp	CTTGTA	14014297	13691409	12863389	93.90%
cvi_1	40bp	ACAGTG	11383586	11272575	10199733	90.40%
cvi_2	40bp	AGTCAA	14068189	13833037	12843468	92.80%
cvi_3	40bp	AGTTCC	11858521	11631134	10763553	92.50%
124_1	40bp	GCCAAT	13378384	13174711	12105362	91.80%
124_2	40bp	ATGTCA	14084336	13869415	12996121	93.70%
124_3	40bp	CCGTCC	12787345	12635141	11686710	92.40%
242_1	40bp	CAGATC	11670732	11317597	10402883	91.90%
242_2	40bp	GTCCGC	15417493	15060348	13863126	92.00%
242_3	40bp	GTGAAA	10743690	10213998	9496429	92.90%

Table S4: Primers used in this study

Primer name	Sequence (5' -> 3')	Target dynamic CG	Purpose
CLP27	CCCTTCTTAAATCARCAACAAACCATC	Chr2:13868028	Forward BS primer
CLP28	AGTATAAAGTAAGGTGYTYGAGTAGTAGT	Chr2:13868028	Reverse BS primer
CLP33	CATCTCACTACATRCATTCTCCARA	Chr2:14019162	Forward BS primer
CLP34	GYATTGAAAGATTATTGAGAAGGAG	Chr2:14019162	Reverse BS primer
CLP35	ATTGGAGATGGTAAGAAGGATGGTT	Chr1:8895443	Forward BS primer
CLP36	AACTAACATACCCTRACAACAACRCA	Chr1:8895443	Reverse BS primer
CLP39	ATGATYAAGAAGAAGTGTGAAGTGAATG	Chr1:7030930	Forward BS primer
CLP40	CAACTTCTTTATCATCCATCTCTTTCTTC	Chr1:7030930	Reverse BS primer
